# Characterization of the *Neurospora crassa* Cell Fusion Proteins, HAM-6, HAM-7, HAM-8, HAM-9, HAM-10, AMPH-1 and WHI-2

**DOI:** 10.1371/journal.pone.0107773

**Published:** 2014-10-03

**Authors:** Ci Fu, Jie Ao, Anne Dettmann, Stephan Seiler, Stephen J. Free

**Affiliations:** 1 Department of Biological Sciences, SUNY University at Buffalo, Buffalo, New York, United States of America; 2 Institute for Biology II, Albert-Ludwigs University Freiburg, Freiburg, Germany; 3 Freiburg Institute for Advanced Studies (FRIAS), Albert-Ludwigs University Freiburg, Freiburg, Germany; Oregon State University, United States of America

## Abstract

Intercellular communication of vegetative cells and their subsequent cell fusion is vital for different aspects of growth, fitness, and differentiation of filamentous fungi. Cell fusion between germinating spores is important for early colony establishment, while hyphal fusion in the mature colony facilitates the movement of resources and organelles throughout an established colony. Approximately 50 proteins have been shown to be important for somatic cell-cell communication and fusion in the model filamentous fungus *Neurospora crassa*. Genetic, biochemical, and microscopic techniques were used to characterize the functions of seven previously poorly characterized cell fusion proteins. HAM-6, HAM-7 and HAM-8 share functional characteristics and are proposed to function in the same signaling network. Our data suggest that these proteins may form a sensor complex at the cell wall/plasma membrane for the MAK-1 cell wall integrity mitogen-activated protein kinase (MAPK) pathway. We also demonstrate that HAM-9, HAM-10, AMPH-1 and WHI-2 have more general functions and are required for normal growth and development. The activation status of the MAK-1 and MAK-2 MAPK pathways are altered in mutants lacking these proteins. We propose that these proteins may function to coordinate the activities of the two MAPK modules with other signaling pathways during cell fusion.

## Introduction

Cell-to-cell fusion between vegetative cells plays a critical role in the life cycles of the filamentous fungi. The fusion between germinating conidia allows the cells to share resources and helps them to establish a colony [1]–[4]. As the fungal colony matures, cell fusion is important for the movement of resources throughout the colony, a prerequisite for asexual and sexual development. In the model filamentous fungus, *Neurospora crassa*, cell-to-cell fusion plays an important role during colony establishment, as well as during conidiation (asexual development) and protoperithecium formation (sexual development) [5]–[7]. During colony establishment, fusion between germinating conidia occurs between specialized cells called conidial anastomosis tubes (CATs), which are morphologically and physiologically distinct from germ tubes [7], [8]. Germ tubes are wider and exhibit negative chemotrophic interactions, while CATs are thinner and exhibit chemotrophic attraction towards each other [8]. Mutants that are defective in cell fusion can’t form an interconnected hyphal network to support nutrient transport within the colony [1], [9]. During the *N. crassa* asexual life cycle, wild type colonies transport nutrients from a vegetative hyphal network into the growing aerial hyphae, which generate conidia (asexual spores). Cell fusion mutants are defective in producing the long aerial hyphae typical of wild type cells. They produce short aerial hyphae, which give a “flat” carpet-like conidiation phenotype. [10]. Cell fusion is also important for the *N. crassa* sexual life cycle. Cell fusion mutants are female sterile, and this may be because the efficient transport of amino acids and other nutrients from a vegetative hyphal network into the developing protoperithecia is needed to support sexual development.

Various groups have defined approximately 50 genes required for cell-cell communication and fusion in *N. crassa* [9]–[17]. Many of these cell fusion genes encode components of the MAK-1 and MAK-2 mitogen-activated protein kinase (MAPK) signal transduction pathways [1], [18]–[24], which are homologous to the yeast cell wall integrity (CWI) and pheromone response cascades, respectively [25]–[27]. MAK-2 and HAM-1/SO, a protein of unknown molecular function, display oscillatory recruitment to opposing cell tips during CAT communication, suggesting that the chemotrophic interactions between two CATs are coordinated by the MAK-2/SO Ping-Pong signaling behavior [23], [28]. NRC-1 and MEK-2, the upstream MAPKKK and MAPKK in MAK-2 pathway, were also found to have the oscillatory signaling behavior during cell fusion [24]. The *N. crassa* MAK-1 CWI pathway initiates through a set of transmembrane sensors. Signals are integrated by the small GTPase RHO1, which activates a conserved mitogen-activated protein kinase (MAPK) cascade through its interaction with protein kinase C [29]–[32]. In addition to its function in cell wall stress integration, the CWI pathway is also a central component of the cell-cell communication machinery. The functional relationship between the two signaling pathways during intercellular communication is poorly understood, but evidence for cross-talk between MAK-1 and MAK-2 is provided by work on the striatin interacting phosphatase and kinase (STRIPAK) complex [33], [34]. Its subunits, HAM-2, HAM-3, HAM-4, MOB-3, PP2A and PPG-1, are all required for cell-cell communication [9], [13], [35], [36]. Phosphorylation of MOB-3 by MAK-2 is required for nuclear localization of MAK-1 in vegetative hyphae, suggesting that MAK-1-dependent expression of cell fusion genes may be required for establishing cell-cell communication competence.

Among the identified cell fusion genes, there were six genes whose functions were largely uncharacterized. To better understand how cell-to-cell fusion is regulated, these six genes, *ham-6*, *ham-8*, *ham-9*, *ham-10*, *amph-1*, and *whi-2* have been further characterized. The expression patterns, intracellular locations, and how the loss of these genes affects the activation status of the MAK-1 and MAK-2 pathway were examined. A seventh gene, *ham-7*, which has been shown to function as a sensor for the MAK-1 pathway [31], was included in the analysis to examine the cell type expression pattern and cellular location of the HAM-7 sensor. In further characterizing these genes, we demonstrate here that HAM-6, HAM-7 and HAM-8 function as upstream elements in the pathway regulating MAK-1 kinase activity during cell fusion. We further demonstrate that HAM-9, HAM-10, AMPH-1 and WHI-2 are proteins with general functions in regulating *N. crassa* growth, and we suggest that HAM-9, HAM-10, and WHI-2 may provide cross-talk between the two MAP kinase pathways and other signaling cascades during cell fusion.

## Materials and Methods

### Strains, media and growth conditions

The strains used in this study are listed in Table 1. Wild type *A* (FGSC#2489), wild type *a* (FGSC#4200), *his-3 A* (FGSC#6103), *histone-1-gfp* (FGSC#9518) [37], *lifeact-rfp* (FGSC#10592) [38], *β-tubulin-gfp* (FGSC#9520) [37], and *mak-1-gfp* (FGSC#10299) [39] were obtained from Fungal Genetics Stock Center (Kansas City, MO). The *Δham-6* (NCU02767), *Δham-7* (NCU00881), *Δham-8* (NCU02811), *Δham-9* (NCU07389), *Δham-10* (NCU02833), *Δamph-1* (NCU01069) and *Δwhi-2*(NCU10518) strains were obtained from the FGSC *N. crassa* single gene deletion mutant library [40]. All other strains used in this study were either obtained through transformation experiments or mating. The presence of the gene deletion in all of the deletion mutant strains was verified by PCR. The growth media and growth condition for regular strain maintenance, mating, and for conidial anastomosis tube (CAT) formation are available through the FGSC website (www.fgsc.net). The screening procedure to identify cell fusion mutants was performed as previously described [9]. Deletion mutant isolates were further characterized by co-segregation experiments to assess whether the gene deletion (marked by the presence of the hygromycin resistance gene cassette) was responsible for the mutant phenotype [9], [40]. Deletion mutants showing co-segregation of hygromycin resistance with the cell fusion mutant phenotypes were verified by complementation experiments.

**Table 1:** Strains used in this study.

Strain	Genotype	Strain source
*Wild-type 74*	*OR23-1 V Mat A*	FGSC#2489
*Wild-type ORS*	*SL6 Mat a*	FGSC#4200
*his-3 A*	*his-3 Mat A*	FGSC#6103
*his-3 a*	*his-3 Mat a*	This study
*histone-1-gfp*	*Pccg-1::hH1+-sgfp+::his-3+ Mat A*	FGSC#9518
*lifeact-rfp*	*Pccg-1::lifeact-rfp::bar+*	FGSC#10592
*β-tubulin-gfp*	*Pccg-1::Bml+-sgfp+::his-3+ Mat A*	FGSC#9520
*Sgfp*	*Pccg-1::sgfp::his-3+ Mat A*	This study
*Rfp*	*Pccg-1::rfp::his-3+ Mat A*	This study
*grp-sgfp*	*Pccg-1::grp-sgfp::his-3+ Mat A*	This study
*rfp-vps-52*	*Pccg-1::rfp-vps-52::his-3+ Mat A*	This study
*arg-4-gfp*	*Pccg-1::arg-4-sgfp::his-3+ Mat A*	This study
*rfp-vam-3*	*Pccg-1::rfp-vam-3::his-3+ Mat A*	This study
*Δham-6 A/a*	*Δham-6::hygR Mat A/Δham-6::hygR Mat a*	FGSC#16993/16903
*Δham-6; his-3*	*Δham-6::hygR, his-3-*	This study
*Δham-7 A/a*	*Δham-7::hygR Mat A/Δham-7::hygR Mat a*	FGSC#13776/13775
*Δham-7; his-3*	*Δham-7::hygR, his-3-*	This study
*Δham-8 A/a*	*Δham-8::hygR Mat A/Δham-8::hygR Mat a*	This study/FGSC#17225
*Δham-8; his-3*	*Δham-8::hygR, his-3-*	This study
*Δham-9 A/a*	*Δham-9::hygR Mat A/Δham-9::hygR Mat a*	This study/FGSC#19549
*Δham-9; his-3*	*Δham-9::hygR, his-3-*	This study
*Δham-10 A/a*	*Δham-10::hygR Mat A/Δham-10::hygR Mat a*	FGSC#21396/21395
*Δham-10; his-3*	*Δham-10::hygR, his-3-*	This study
*Δamph-1 A/a*	*Δamph-1::hygR Mat A/Δamph-1::hygR Mat a*	FGSC#12550/12549
*Δamph-1; his-3*	*Δamph-1::hygR, his-3-*	This study
*Δwhi-2 A/a*	*Δwhi-2::hygR Mat A/Δwhi-2::hygR Mat a*	FGSC#21578/This study
*Δwhi-2; his-3*	*Δwhi-2::hygR, his-3-*	This study
*mak-1-sgfp*	*Pccg-1::mak-1-sgfp::bar+*	FGSC10299
*mak-1-sgfp; Δham-6*	*Pccg-1::mak-1-sgfp::bar+; Δham-6::hygR*	This study
*mak-1-sgfp; Δham-7*	*Pccg-1::mak-1-sgfp::bar+; Δham-7::hygR*	This study
*mak-1-sgfp; Δham-8*	*Pccg-1::mak-1-sgfp::bar+; Δham-8::hygR*	This study
*mak-1-sgfp; Δham-9*	*Pccg-1::mak-1-sgfp::bar+; Δham-9::hygR*	This study
*mak-1-sgfp; Δham-10*	*Pccg-1::mak-1-sgfp::bar+; Δham-10::hygR*	This study
*mak-1-sgfp; Δamph-1*	*Pccg-1::mak-1-sgfp::bar+; Δamph-1::hygR*	This study
*mak-1-sgfp; Δwhi-2*	*Pccg-1::mak-1-sgfp::bar+; Δwhi-2::hygR*	This study
*mak-2-sgfp*	*Pccg-1::mak-2-sgfp::his-3+*	This study
*mak-2-sgfp; Δham-6*	*Pccg-1::mak-2-sgfp::his-3+; Δham-6::hygR*	This study
*mak-2-sgfp; Δham-7*	*Pccg-1::mak-2-sgfp::his-3+; Δham-7::hygR*	This study
*mak-2-sgfp; Δham-8*	*Pccg-1::mak-2-sgfp::his-3+; Δham-8::hygR*	This study
*mak-2-sgfp; Δham-9*	*Pccg-1::mak-2-sgfp::his-3+; Δham-9::hygR*	This study
*mak-2-sgfp; Δham-10*	*Pccg-1::mak-2-sgfp::his-3+; Δham-10::hygR*	This study
*mak-2-sgfp; Δamph-1*	*Pccg-1::mak-2-sgfp::his-3+; Δamph-1::hygR*	This study
*mak-2-sgfp; Δwhi-2*	*Pccg-1::mak-2-sgfp::his-3+; Δwhi-2::hygR*	This study
*so-sgfp*	*Pccg-1::so-sgfp::his-3+*	This study
*so-sgfp; Δham-6*	*Pccg-1::so-sgfp::his-3+; Δham-6::hygR*	This study
*so-sgfp; Δham-7*	*Pccg-1::so-sgfp::his-3+; Δham-7::hygR*	This study
*so-sgfp; Δham-8*	*Pccg-1::so-sgfp::his-3+; Δham-8::hygR*	This study
*so-sgfp; Δham-9*	*Pccg-1::so-sgfp::his-3+; Δham-9::hygR*	This study
*so-sgfp; Δham-10*	*Pccg-1::so-sgfp::his-3+; Δham-10::hygR*	This study
*so-sgfp; Δamph-1*	*Pccg-1::so-sgfp::his-3+; Δamph-1::hygR*	This study
*so-sgfp; Δwhi-2*	*Pccg-1::so-sgfp::his-3+; Δwhi-2::hygR*	This study
*HA-ham-6; Δham-6*	*Pham-6::HA-ham-6::his-3+; Δham-6::hygR*	This study
*HA-ham-7; Δham-7*	*Pham-7::HA-ham-7::his-3+; Δham-7::hygR*	This study
*HA-ham-8; Δham-8*	*Pham-8::HA-ham-8::his-3+; Δham-8::hygR*	This study
*HA-ham-9; Δham-9*	*Pham-9::HA-ham-9::his-3+; Δham-9::hygR*	This study
*HA-amph-1; Δamph-1*	*Pamph-1::HA-amph-1::his-3+; Δamph-1::hygR*	This study
*HA-whi-2; Δwhi-2*	*Pwhi-2::HA-whi-2::his-3+; Δwhi-2::hygR*	This study
*ham-8-sgfp; Δham-8*	*Pccg-1::ham-8-sgfp::his-3+; Δham-8::hygR*	This study
*rfp-ham-8; Δham-8*	*Pccg-1::rfp-ham-8::his-3+; Δham-8::hygR*	This study
*rfp-ham-10; Δham-10*	*Pccg-1::rfp-ham-10::his-3+; Δham-10::hygR*	This study
*rfp-amph-1; Δamph-1*	*Pccg-1::rfp-amph-1::his-3+; Δamph-1::hygR*	This study

### Plasmid construction and expression of tagged proteins

Plasmids used in this study are listed in Table S1. The primers used to construct GFP-tagged, RFP-tagged and HA-tagged protein constructs are listed in Table S2. GFP and dsRed RFP fusion proteins were generated using the pMF272 and pMF334 vectors [37], [41] and were expressed under the control of *ccg-1* promoter at the *his-3* locus [42].

HA-tagged proteins (HA-HAM-6, HA-HAM-8, HA-HAM-9, HA-AMPH-1 and HA-WHI-2) were cloned using the vectors pBM60 and pBM61 [43]–[45] and were expressed under the control of their own promoters at the *his-3* locus. To identify sites for HA tagging, the protein sequences for HAM-6, HAM-8, HAM-9, AMPH-1 and WHI-2 were used to generate multiple sequence alignments with homologous proteins from other fungi [46]. Protein sequences were also analyzed by the online program Globplot to predict protein structural information [47]. Non-conserved protein sequence regions, which were predicted to be exposed, were chosen for HA tagging. For *ham-6*, *ham-8* and *ham-9*, the HA tag coding sequence was inserted immediately before the stop codon. For *amph-1*, the HA tag coding sequence was inserted between the third and the fourth amino acid codons. For *whi-2*, the peptide sequence VPVDPASGA (amino acid 118–126) was replaced with the HA tag. The cloning of these HA-tagged protein constructs involved two PCR amplification steps. PCR primers were used to amplify approximately 1,500 bp of 5′ UTR sequence, the coding sequence upstream of the HA tag insertion site, and the HA tag coding sequence. A second set of primers were used to amplify the HA tag coding sequence, the rest of the coding sequence of the gene, and the approximately 500 bp 3′ UTR sequence. The two PCR products were then mixed together and used as templates to amplify the entire HA-tagged gene. The primers designed for the two ends of the genes contained an added restriction enzyme site to allow the insertion of the amplified DNA into the multicloning sites of pBM60 or pBM61. An HA-tagged version of HAM-7 with its endogenous promoter was generated by Retrogen Inc. (San Diego, CA). The DNA sequence encoding amino acids 190–198 (YTINILESG), which are located immediately in front of the GPI anchor addition site, were replaced by the HA tag sequence (YPYDVPDYA) in the HA-tagged HAM-7.

Plasmids pgrp-GFP, pRFP-vps-52, parg-4-GFP and pRFP-vam-3 were obtained from the FGSC as fluorescent protein markers for ER, Golgi, mitochondria, and vacuoles respectively [48], and used in co-localization experiments. Plasmids pso-GFP and pmak-2-GFP were kind gifts from Dr. Louise Glass’s lab, and were used to study the MAK-2 signal transduction pathway [23].

### Analysis of HA-tagged protein expression

To examine the expression of HA-tagged proteins in vegetative hyphae, cultures were grown in 100 ml liquid Vogel’s sucrose medium in shaking Erlenmeyer flasks at room temperature for 36 to 48 hours. To examine the expression of HA-tagged proteins in germ tubes and CATs, conidia were used to inoculate 16 ml of Vogel’s sucrose medium at a titer of 10^6^ per ml and grown in 100×15 mm Petri dishes at 34°C without agitation for 4 hours to allow the formation of germ tubes and CATs [8]. Cells were harvested by filtration using a Büchner funnel and ground in liquid nitrogen. Protein extraction buffer [100 mM Tris/HCL pH 7.4, 1% (w/v) SDS; supplemented with 1X protease cocktail (P-8340 Sigma-Aldrich, St. Louis, MO)] was added, and the cell extracts were collected after centrifugation.

For Western blot analysis of HA-tagged proteins, the protein concentration of the cell extracts was determined by using the DC protein assay kit (BioRad, Hercules, CA). Samples containing 60 µg of protein were subjected to SDS-PAGE and transferred to nitrocellulose membrane. The nitrocellulose membranes were then subjected to Ponceau S red (Sigma-Aldrich, St. Louis, MO) staining to verify equal loading of different protein samples. Western blot experiments with mouse monoclonal anti-HA (Covance, Princeton, NJ) and rabbit anti-mouse IgG-HRP (Sigma-Aldrich, St. Louis, MO) were used to assess the level of protein expression. Chemiluminescent signal was detected by using a ChemiDoc XRS+ System and images were analyzed with Image Lab Software (BioRad, Hercules, CA).

### Evaluation of MAK-1 and MAK-2 phosphorylation status

Polyclonal antibody directed against the phosphorylated activation site in common on MAK-1 and MAK-2 was used to evaluate their activation status. To examine the status of the pathways in vegetative hyphae, liquid *N. crassa* cultures were grown at room temperature and harvested by filtration using a Büchner funnel [31]. In some experiments, vegetative cultures were subjected to 8 mM H_2_O_2_ for 10 minutes just prior to being harvested to determine if the pathways could be activated by oxidative stress. To study the MAK-1 and MAK-2 phosphorylation status in germ tubes and CATs, conidia were grown for four hours under the conditions described above to allow germ tube and CAT formation. Germlings were harvested by gently scraping them off the culture dishes and collected on a Büchner funnel. The harvested vegetative hyphae samples and germ tubes/CATs samples were ground to a fine powder in a mortar and pestle in liquid nitrogen. Protein extraction for the analysis of the MAK-1 and MAK-2 phosphorylation status was performed as described by Maddi et al. [31].

### Preparation of cells expressing HA-tagged proteins for immunolocalization

For each HA-tagged strain, six Petri dishes containing 16 ml of conidial suspension (10^6^ conidia/ml) were grown at 34°C for 4 hours to allow germ tube and CAT formation. Cells were harvested by gently scraping them off Petri dishes, and were collected by centrifugation at 6000×g for 5 minutes. Cells were transferred into a microcentrifuge tube and fixed in PBS (phosphate buffered saline) with 3.7% formaldehyde for 15 minutes. After washing with PBS, the samples were incubated for 30 minutes in PBS containing 1 mg/ml of Novozyme 234 (InterSpex Products Inc., Foster City, CA) and 1% bovine serum albumin to digest the cell wall [49], [50]. Following the cell wall digestion step, the cells were collected by centrifugation and washed with PBS. Cell samples were then incubated in permeabilization buffer (PBS with 1% BSA and 0.5% Triton-X 100) for 5 minutes to permeabilize the membrane. After the membrane permeabilization step, the cells were collected and washed with PBS. Cell samples were then incubated overnight at 4°C in mouse monoclonal anti-HA primary antibody (Covance, Princeton, NJ) used at a 1∶200 dilution in PBS with 1% BSA. After the primary antibody incubation, samples were washed in PBS three times and then incubated for 2 hours in Alexa Fluor 488-conjugated goat anti-mouse secondary antibody (Life Technologies, Carlsbad, CA) used at a 1∶100 dilution in PBS with 1% BSA. After three washes, cell samples were used for microscopic observation. All of the incubation buffers and washing buffers used in this assay were supplemented with 1X protease inhibitor cocktail and 1 mM PMSF (Sigma Aldrich, St. Louis, MO).

### Live cell imaging for the quantification of CATs

Live-cell imaging was performed using an inverted microscope Diaphot-TMD inverted microscope (Nikon, Japan) to quantify CAT fusion activity for wild type and mutant strains as previously described [8], [51]. In brief, 1 ml of fresh conidia at a density of 10^6^ per ml were grown in Petri dishes (35×10 mm) at 34°C. Benomyl was added to distinguish CATs from germ tubes. This is because benomyl inhibits the formation of long germ tubes but not CAT formation or fusion [52], [53]. After 4 hours of incubation, the cells were examined under the microscope for the presence of CATs and CAT fusion. For each sample, five random images were captured for later quantification of CAT fusion activity. The frequency of cell fusion observed in the mutant samples was compared to the wild type cell fusion frequency to obtain a relative CAT fusion activity for each of the mutants.

### Sample preparation and confocal microscopy

For live-cell imaging of CAT formation, an inverted agar block method was adapted for this study [54]. A 1 ml aliquot of conidia expressing GFP-tagged protein or RFP-tagged protein (10^6^ conidia per ml) was placed in a fluorodish (World Precision Instruments, FL). A thick agar block was then placed in the middle of the fluorodish to facilitate attachment of the conidia to the surface of the cover glass bottom. Benomyl was added in both the liquid medium and the agar block to inhibit germ tube formation. The cells were observed between 3 and 6 hours to follow the formation of CATs.

For imaging of germ tubes and hyphal cells expressing fluorescent protein constructs, conidia (10^5^ conidia per ml) were grown in Vogel’s sucrose liquid medium for 6 to 8 hours. Cell samples were placed on slides for immediate microscopic observation.

To examine the nuclear localization of MAK-1-GFP and MAK-2-GFP in germ tubes/CATs, conidia (10^6^ per ml) were grown in Vogel’s sucrose liquid medium at 34°C for 4 hours to allow conidia germination and CAT formation. Cell samples were then collected, fixed, digested with Novozyme, and permeabilized following the procedure described above. After membrane permeabilization, cells were treated with 1 mg/ml RNAase for 30 minutes at room temperature to digest RNA. Cells were then washed once with PBS and stained with propidium iodide (5 µg/ml propidium iodide in PBS) for 20 minutes [55]. After three washing steps, cells were placed on slides for microscopic observation.

Confocal laser scanning microscopy was performed using a Zeiss LSM 710 Confocal Microscope (Carl Zeiss, Inc., Thornwood, NY). Plan-Apochromat 63×/1.40 Oil DIC M27 objective or Plan-Apochromat 40×/1.3 Oil DIC M27 objective lenses were used for imaging. Excitation wavelength and detection wavelength were set up either according to references [37], [41], [56] or by using the smart setup program of the ZEN2012 image capture software. GFP images were collected at 493 to 598 nm with excitation at 488 nm. RFP images were collected at >570 nm with excitation at 558 nm. When imaging samples with both GFP and RFP signals, the images were collected at 493 to 539 nm and at 554 to 703 nm with excitation at 490 nm and 514 nm. When examining GFP protein samples that have been stained with propidium iodide, the images were collected at 499 to 560 nm and at 572 to 719 nm with excitation at 488 nm and 535 nm. The images were collected sequentially using either a line sequential scanning mode or plane scanning mode. Bright-field images were captured with a transmitted light detector. Time-lapse imaging was performed to evaluate Ping-Pong signaling at time intervals of 10 s to 60 s for periods up to 10 minutes. Images were analyzed with image processing software ZEN lite and Image J.

For the detection of HA-tagged proteins, anti-HA primary antibody was used in conjunction with Alexa Fluor 488 conjugated secondary antibody. Immunofluorescent images were collected at 497 to 622 nm with excitation at 488 nm.

## Results

### Characterization of cell fusion mutants reveals two functional mutant groups

**Figure 1:**
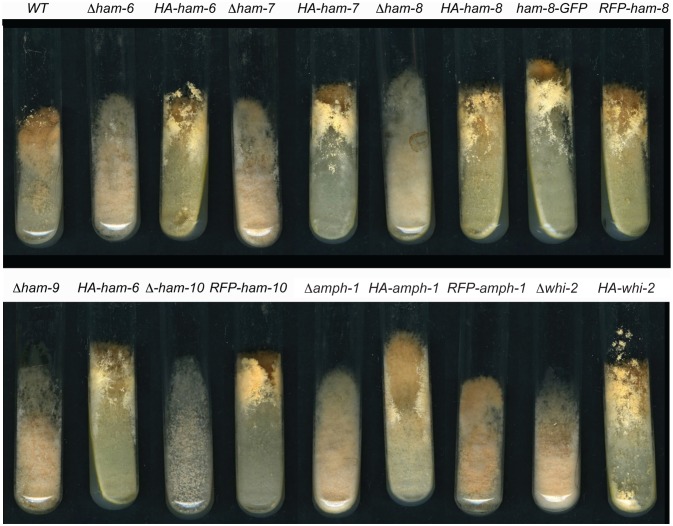
Strains used in this study. Slants containing Vogel’s sucrose medium were inoculated with different strain isolates and grown for 4 days. Strains shown in the top panel from left to right include wild type (WT), *Δham-6*, *Δham-6* transformed with *HA-ham-6*, *Δham-7*, *Δham-7* transformed with *HA-ham-7*, *Δham-8*, *Δham-8* transformed with *HA-ham-8*, *Δham-8* transformed with *ham-8-GFP*, and *Δham-8* transformed with *RFP-ham-8*. The bottom panel shows *Δham-9*, *Δham-9* transformed with *HA-ham-9*, *Δham-10*, *Δham-10* transformed with *RFP-ham-10*, *Δamph-1*, *Δamph-1* transformed with *HA-amph-1*, *Δamph-1* transformed with *RFP-amph-1*, *Δwhi-2*, and *Δwhi-2* transformed with *HA-whi-2*.

**Figure 2:**
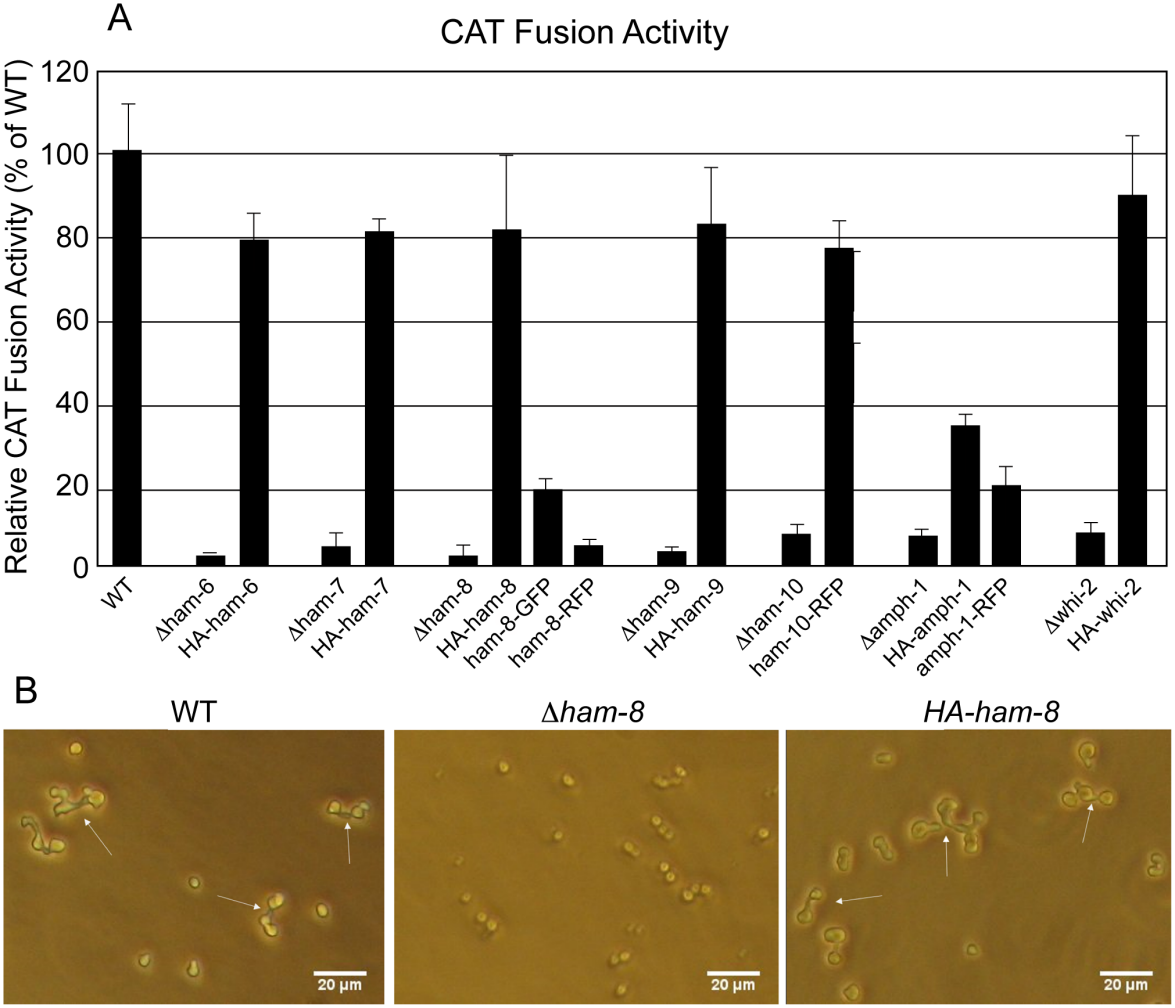
Complementation of CAT fusion activities by different HA, GFP and RFP tagged proteins. A) The levels of CAT fusion activity for the gene deletion mutants and for transformants expressing a tagged version of the deleted gene are shown as a percentile of the cell fusion activity for wild type CATs. B) Photograph of CAT fusion activities in wild type (WT), *Δham-8*, and *Δham-8* transformed with *HA-ham-8*. Arrows point to examples of CAT fusion in the wild-type and *Δham-8* transformed with *HA-ham-8* panels.

A screening of plates 110 to 120 from the single gene deletion library identified a new cell fusion gene, *whi-2* (NCU10518), which is a predicted homolog of yeast stress response factor protein Whi2p [57], [58]. We confirmed that the cell-fusion defect and hygromycin resistance co-segregated. Moreover, ectopic expression of HA-WHI-2 at the *his-*3 locus complemented the *Δwhi-2* mutant phenotypes (Figure 1 and 2), demonstrating that the loss of *whi-2* was responsible for the mutant defects. An examination of the conidial morphology of *Δwhi-2* revealed a conidial phenotype similar to that of the *Δham-10* and *Δamph-1* mutants (Figure S1). Instead of making mature macroconidia, *Δham-10*, *Δamph-1* and *Δwhi-2* produced chains of macroconidia that stopped development at the major constriction stage (Figure S1) [59]. *Δham-10* produced fewer conidia than *Δamph-1* and *Δwhi-2*. In contrast, *Δham-6*, *Δham-7*, *Δham-8* and *Δham-9* generated normal macroconidia (Figure S1).

All of the cell fusion mutants had a flat conidiation phenotype, which is due to a defect in the generation of long aerial hyphae (Figure 1). The phenotypic differences between the *Δham-6*, *Δham-7*, *Δham-8* and *Δham-9* group of mutants, and the *Δham-10*, *Δamph-1* and *Δwhi-2* mutants suggest that there are functional differences between the proteins encoded by these two groups of cell fusion genes.

### HAM-6, HAM-7 and HAM-8 are specifically expressed in germ tubes/CATs

**Figure 3:**
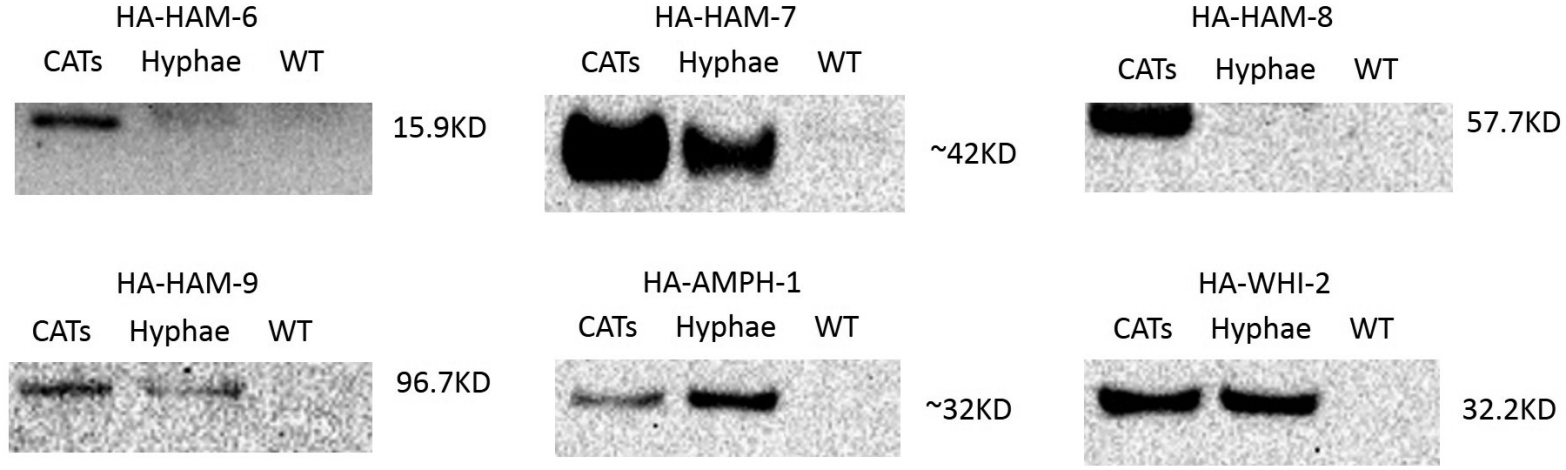
Western blot analyses of HA-tagged proteins’ expression patterns. Western blot analyses using anti-HA antibody were performed to detect HA-HAM-6, HA-HAM-7, HA-HAM-8, HA-HAM-9, HA-AMPH-1 and HA-WHI-2 protein levels in four hour germlings (CATs lane) and vegetative hyphae (Hyphae lane). The HA-tagged cell fusion proteins were regulated by their own promoters. Protein samples from wild type germ tubes/CATs were loaded as negative control (WT lane) for each Western blot analysis.

To examine the cell type expression pattern for these cell fusion proteins, we generated HA-tagged versions that were expressed at the *his-3* locus under the control of their own promoters. The HA-tagged proteins fully rescued the mutant developmental and CAT fusion defects of *Δham-6*, *Δham-7*, *Δham-8*, *Δham-9* and *Δwhi-2* (Figures 1 and 2) [9]. The HA-tagged version of AMPH-1 provided only a partial rescue of *Δamph-1* (32.3% of the wild type cell fusion level) (Figures 1 and 2). Western blot analysis was performed to examine the size and expression patterns of the HA-tagged proteins (Figure 3). The predicted MW (molecular weight) for HAM-6, HAM-7, HAM-8, HAM-9, AMPH-1 and WHI-2 are 15.9 KD, 24.4 KD, 57.7 KD, 96.7 KD, 29.9 KD and 32.2 KD respectively. HAM-9, AMPH-1 and WHI-2 are predicted to be cytosolic proteins, and their HA-tagged proteins gave MWs very close to the predicted MWs. HAM-6 and HAM-8 contain three and four predicted TM (transmembrane) domains respectively, and the measured MWs of their HA-tagged proteins were also very close to their predicted MWs. HAM-7 has been shown to be a GPI-anchored cell wall protein [31]. The measured MW for HA-HAM-7 was 42 KD, 18 KD larger than the predicted MW, which suggests the GPI-anchored cell wall protein is heavily glycosylated.

HA-HAM-6 and HA-HAM-8 displayed a germ tubes/CATs-specific expression pattern, with only a trace amount of expression in vegetative hyphae (Figure 3). HA-HAM-7 was expressed at a very high level in germ tubes and CATs, and at a 5-fold reduced level in vegetative hyphae. In contrast, we determined that HA-HAM-9 and HA-WHI-2 were expressed at about equal level in the germ tubes/CATs and hyphae samples, while HA-AMPH-1 was expressed at higher level in hyphae than in germ tubes/CATs (Figure 3). In summary, these expression experiments support our phenotypic classification of the mutants, and suggest that HAM-6, HAM-7, and HAM-8 form a group of proteins that primarily functions in germlings and during CAT fusion, while HAM-9, WHI-2 and AMPH-1 have general functions during growth and differentiation.

### HAM-7 and HAM-8 are found in a punctate pattern near the tips of germ tubes and CATs

**Figure 4:**
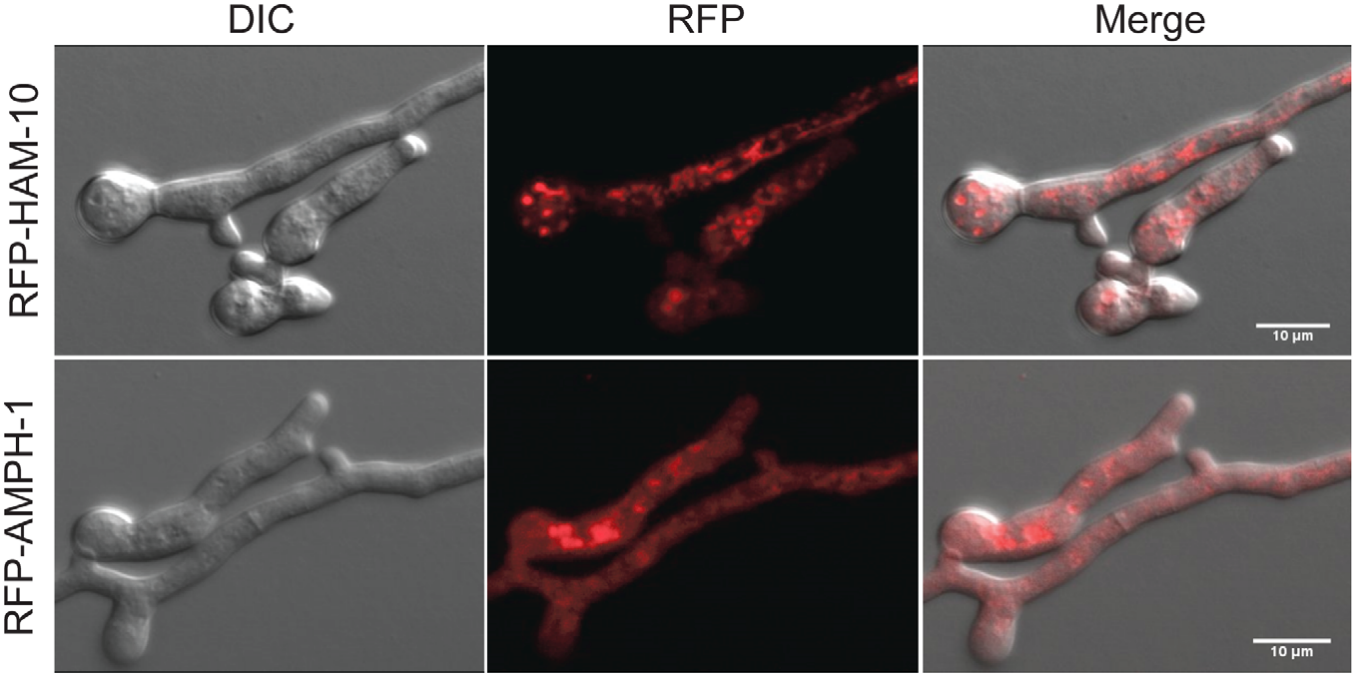
Localization of RFP-HAM-10 and RFP-AMPH-1 in germ tubes/CATs. Confocal microscopic images were taken for CATs expressing RFP-HAM-10 (top row of panels) and RFP-AMPH-1 (bottom row of panels). Images shown from left to right are DIC images, RFP fluorescent images, and merged images.

In order to determine the location of the cell fusion proteins, we expressed them as GFP- and dsRed RFP-tagged constructs under the control of the *ccg-1* promoter in their respective mutant backgrounds (Figure 1). The HAM-9-GFP, RFP-HAM-9, HAM-10-GFP and AMPH-1-GFP fusion proteins failed to rescue the mutant phenotypes. We were unable to detect the GFP and RFP signals from these tagged proteins, suggesting that the tagged proteins were rapidly degraded. HAM-8-GFP and RFP-HAM-8 rescued the *Δham-8* conidiation defects, but failed to restore CAT fusion activity, indicating that the tagged HAM-8 proteins were only partially functional (Figures 1 and 2). Moreover, some of the GFP and RFP signals were detected at large vacuolar-like structures, suggesting that the tagged HAM-8 proteins might have been targeted to the vacuole for degradation, and may not reflect the normal localization for HAM-8 (Figure S2A). Co-localization experiments with marker proteins showed that HAM-8-GFP and the vacuolar marker RFP-VAM-3 showed co-localization (Figure S2B). RFP-AMPH-1 gave a partial rescue on both conidiation phenotype and CAT fusion activity (18.5% of wild type cell fusion level) (Figures 1 and 2), and localized in a punctate pattern, suggestive of being associated with small vesicles (Figure 4). We also detected RFP signal in larger vacuolar-like structures, which may represent RFP entering into vacuoles and being degraded (see below). RFP-HAM-10 was the only fluorescent fusion protein that fully rescued both mutant conidiation phenotype and CAT fusion activity (Figures 1 and 2). RFP-HAM-10 was found in vesicular or vacuolar-like structure in germ tubes and CATs (Figures 4 and S3). Because MAK-2 and SO have been detected in association with small vesicles near the tips of CATs, we asked whether the RFP-HAM-10 and RFP-AMPH-1 co-localized with MAK-2-GFP or SO-GFP (Figures S3 and S4). We did not see evidence for the co-localization of RFP-HAM-10 or RFP-AMPH-1 with either MAK-2-GFP or SO-GFP near the tips of CATs during cell fusion (Figures S3 and S4).

**Figure 5:**
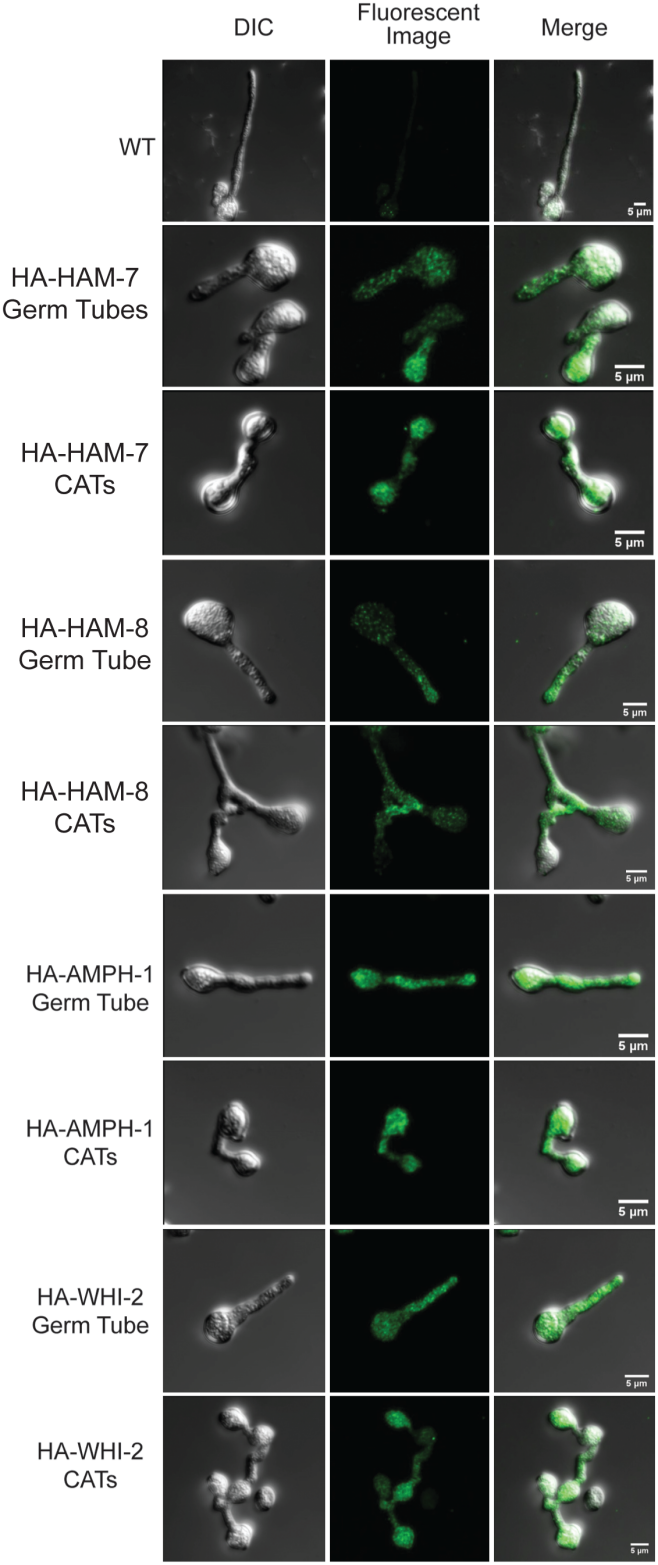
Immunofluorescent localization images for HA-tagged proteins. Anti-HA primary antibody and Alexa Fluor 488-conjugated secondary antibody were used to label HA-tagged protein in fixed germ tubes/CATs. Typical DIC images (left), fluorescent images (middle), and merged images (right) are shown. Images are shown for Wild type (WT) control (top row), *HA-ham-7* transformant germ tubes (row 2), and CATs (row 3), *HA-ham-8* transformant germ tube (row 4) and CATs (row 5), *HA-amph-1* transformant germ tube (row 6) and CATs (row 7), *HA-whi-2* transformant germ tube (row 8) and CATs (bottom row).

The HA-tagged versions of HAM-6, HAM-7, HAM-8, HAM-9, AMPH-1, and WHI-2, expressed under the control of their endogenous promoters, provided an alternative opportunity to examine the location of these proteins in fixed cells. Except for HA-AMPH-1, which was only partially functional, the HA-tagged version of these proteins fully rescued the mutant defects (Figures 1 and 2). We were unable to get immunolocalization data for HA-HAM-6 and HA-HAM-9, which was not surprising because these two proteins were expressed at very low levels (Figure 3). HA-HAM-7 and HA-HAM-8 were localized in a punctate pattern, suggestive of being found in small vesicles or vacuoles (Figures 5A and 5B). The intensity of the fluorescent signal for HA-HAM-8 near the tip region of the germ tubes/CATs was consistently found to be significantly higher than the signal in the rest of the cell.

HA-AMPH-1 was localized in a punctate pattern, suggestive of being associated with small vesicle, and in the cytosol (Figure 5C), consistent with the localization pattern of RFP-AMPH-1 (Figure 4). Significantly, we did not detect HA-AMPH-1 in large vacuolar-like structures, underscoring our suggestion that the GFP and RFP-tagged fusion proteins detected in large vacuolar-like structures have been targeted to the vacuoles for degradation. HA-WHI-2, which is predicted to be a cytosolic protein, also showed localization to what appear to be small vesicles or vacuoles as well as to the cytosol. (Figure 5D).

### Oscillatory recruitment of MAK-2 and SO to cell tips is abolished in cell fusion mutants

**Figure 6:**
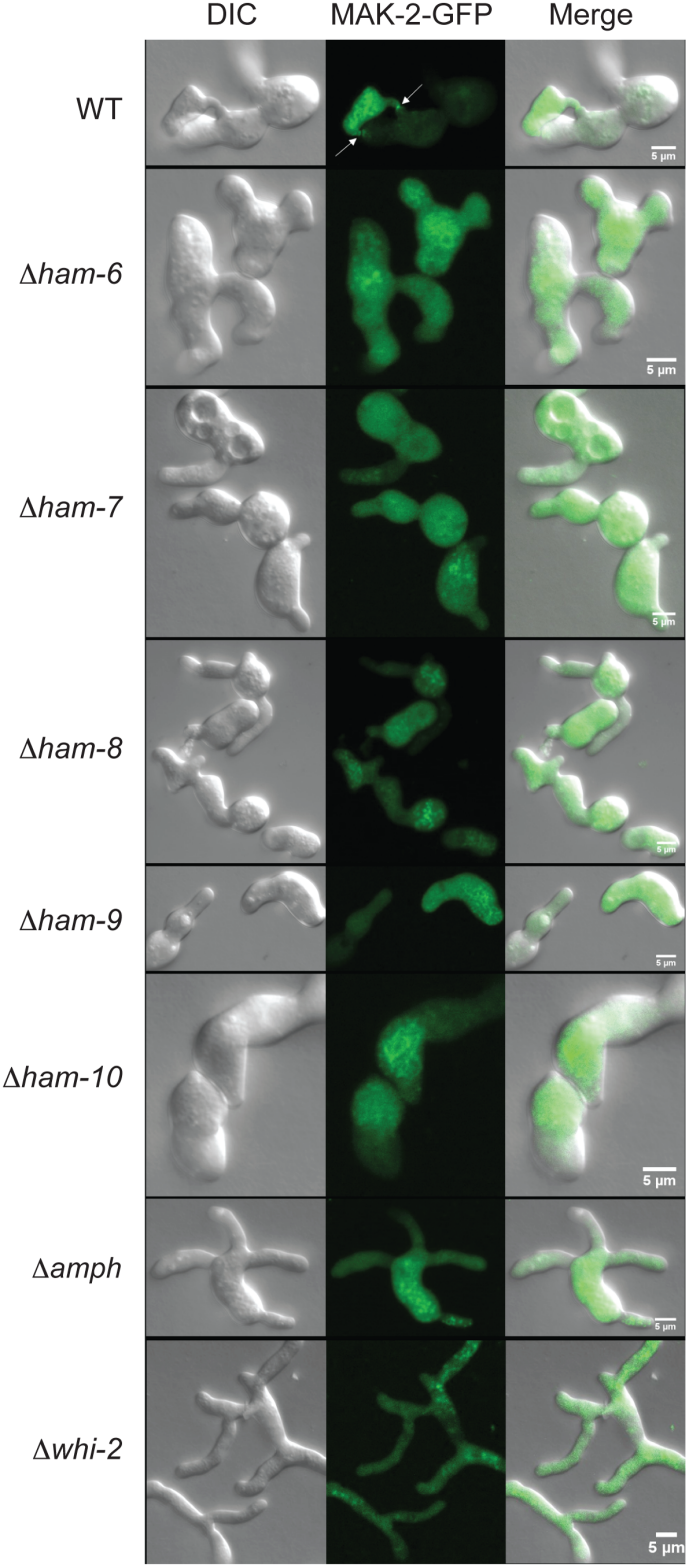
MAK-2-GFP localization in wild type and mutant germ tubes/CATs. MAK-2-GFP expressing wild type (WT) and mutant conidia were grown under CAT induction conditions for 4 hours. DIC images (left column), GFP fluorescent images (middle column), and merged images (right column) are shown. The images show germ tubes/CATs for wild type (WT) (row 1), *Δham-6* (row 2), *Δham-7* (row 3), *Δham-8* (row 4), *Δham-9* (row 5), *Δham-10* (row 6), *Δamph-1* (row 7), and *Δwhi-2* (row 8). The arrows in the WT GFP fluorescent image point to the localization of MAK-2-GFP at the sites of cell fusion.

MAK-2 and SO have been shown to be recruited to the cell tips in an oscillatory fashion during CAT fusion [23]. In an effort to identify whether “Ping-Pong” signaling is disrupted in our cell fusion mutants, we generated MAK-2-GFP-expressing and SO-GFP-expressing *Δham-6*, *Δham-7*, *Δham-8*, *Δham-9*, *Δham-10*, *Δamph-1* and *Δwhi-2* isolates by mating cell fusion mutants with MAK-2-GFP-expressing and SO-GFP-expressing wild type strains of the opposite mating type. Germinating conidia of these mutant strains made CAT-like structures in non-cell fusion contexts at very low frequency. Germ tube germination was not affected in cell fusion mutants (Figure 6). Microscopic examination of these strains showed that MAK-2-GFP and SO-GFP never localized at the tip of germ tubes or CAT-like structures (Figure 6).

**Figure 7:**
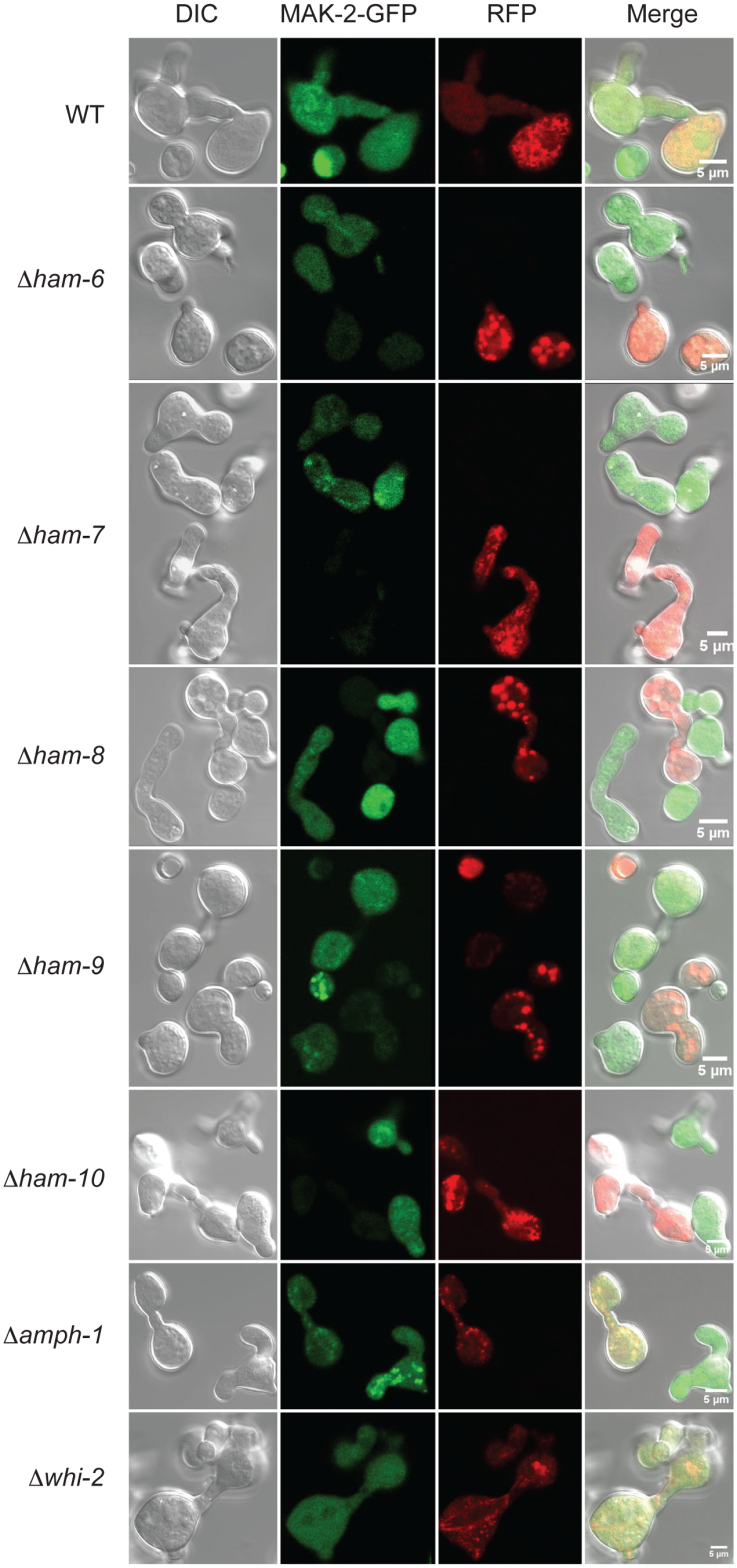
Fusion between MAK-2-GFP-expressing cell fusion mutants and RFP-expressing wild type cells. Conidia samples containing equal number of RFP-expressing wild type conidia and MAK-2-GFP-expressing wild type or mutant conidia were grown under CAT induction conditions for 4 hours. DIC images, GFP fluorescent images, RFP fluorescent images, and merged images for each combination of conidia types are shown in the columns from left to right respectively. Each row shows the images for RFP-expressing wild type conidia mixed with MAK-2-GFP-expressing wild type (WT) (row 1), *Δham-6* (row 2), *Δham-7* (row 3), *Δham-8* (row 4), *Δham-9* (row 5), *Δham-10* (row 6), *Δamph-1* (row 7), and *Δwhi-2* (row 8) conidia. Wild type conidia frequently engaged in cell fusion, while *Δamph-1* and *Δwhi-2* conidia engaged in cell fusion with w conidia at a low frequency.

In separate experiments, mutant conidia expressing MAK-2-GFP and SO-GFP were mixed with an equal number of wild type conidia expressing cytosolic RFP to determine whether the mutant conidia could respond to wild type signals, participate in MAK-2/SO Ping-Pong signaling, and fuse with wild type conidia. We observed that *Δamph-1* and *Δwhi-2* conidia were able to fuse with wild type at a very low frequency while the remaining mutants never fused with wild type. Interestingly, the few *Δamph-1* and *Δwhi-2* conidia that participated in cell fusion with wild type displayed normal macroconidial morphologies (Figure 7), while the typical, abnormally-shaped mutant conidia did not (see Figure S1). Because fusions between wild type and *Δamph-1* or *Δwhi-2* conidia were very rare, we were unable to determine whether the oscillatory recruitment of MAK-2-GFP and SO-GFP to the CAT tip occurred in these germling pairs.

### MAK-1 and MAK-2 phosphorylation status is affected in mutant germ tubes/CATs and vegetative hyphae

**Figure 8:**
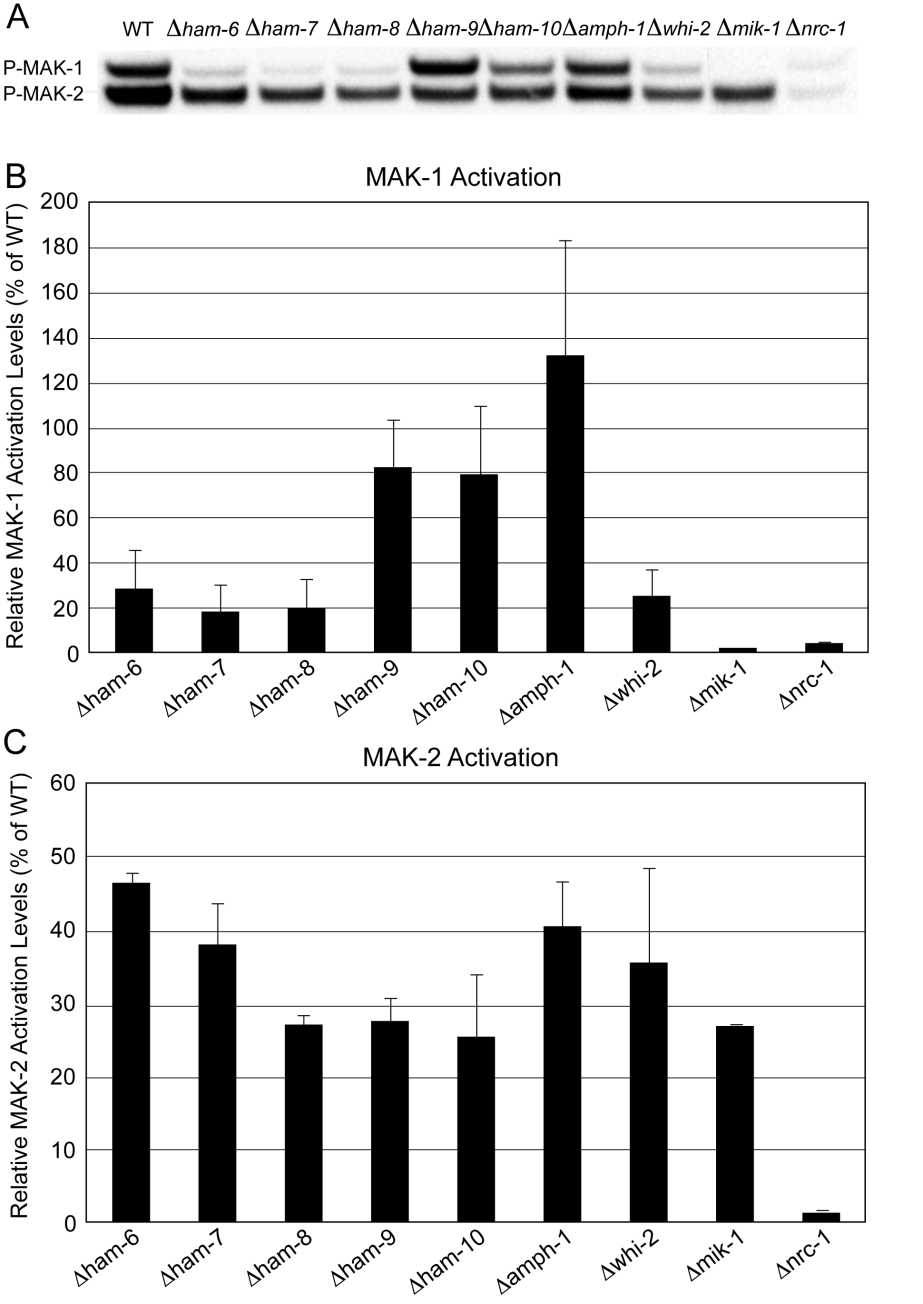
MAK-1 and MAK-2 phosphorylation status in germ tubes/CATs. Western blot analysis using Phospho-p44/42 MAPK antibody was performed to determine MAK-1 and MAK-2 phosphorylation status in wild type (WT) and mutant (*Δham-6*, *Δham-7*, *Δham-8*, *Δham-9*, *Δham-10*, *Δamph-1*, *Δwhi-2*, *Δmik-1*, and *Δnrc-1*) germ tubes/CATS. The positions of the phosphorylated MAK-1 (p-MAK-1) and phosphorylated MAK-2 (p-MAK-2) in the Western blot are noted in the left margin of the figure. B) The relative MAK-1 phosphorylation status in mutant germ tubes/CATs relative to the MAK-1 phosphorylation status in wild type germ tubes/CATs (WT value is set at 100%). C) The relative MAK-2 phosphorylation status in mutant germ tubes/CATs compared to the MAK-2 phosphorylation status in wild type germ tubes/CATs (WT value is set at 100%).

The phosphorylation of MAK-1 and MAK-2 activates the MAP kinases and is required for cell-cell communication and cell fusion. In order to determine whether HAM-6, HAM-7, HAM-8, HAM-9, HAM-10, AMPH-1 and WHI-2 influence the activity of MAK-1 and MAK-2, we looked at the phosphorylation status of both MAPKs in mutant germlings during CAT-inducing conditions (Figure 8). MAK-1 phosphorylation was dramatically reduced in *Δham-6*, *Δham-7*, *Δham-8*, and *Δwhi-2*, and slightly reduced in *Δham-10*. MAK-1 phosphorylation was not reduced in *Δham-9* and *Δamph-1*. These results demonstrate that HAM-6, HAM-7, HAM-8, WHI-2, and perhaps HAM-10 are required for activation of the MAK-1 pathway (Figures 8 and S5). In contrast, MAK-2 phosphorylation was reduced in all cell fusion mutants, which may contribute to the MAK-2/SO signaling defect observed in all of the mutants.

**Figure 9:**
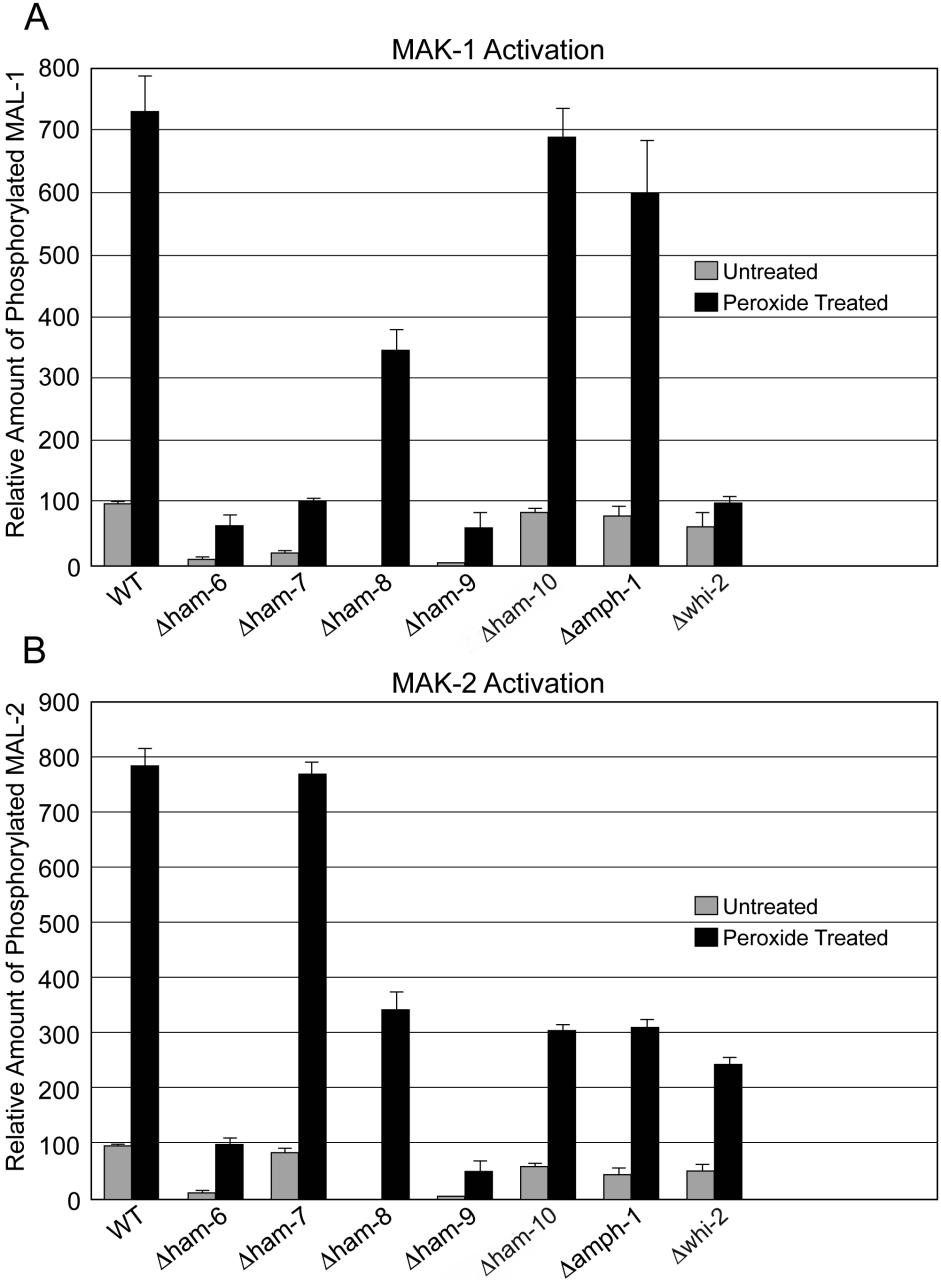
Peroxidase activation of MAK-1 and MAK-2 pathways in cell fusion mutant vegetative hyphal cells. Western blot analyses using Phospho-p44/42 MAPK antibody were performed to evaluate MAK-1 and MAK-2 activation in wild type (WT) and mutant vegetative hyphal cells in response to peroxidase treatments. Quantitative analyses of the Western blots were performed to determine the levels of phosphorylated MAK-1 and MAK-2 in non-stressed and oxidative-stressed samples (wild type, *Δham-6*, *Δham-7*, *Δham-8*, *Δham-9*, *Δham-10*, *Δamph-1*, and *Δwhi-2*). A) MAK-1 activation in wild type and mutants in response to peroxidase treatment. B) MAK-2 activation in wild type and mutants in response to peroxidase treatment. The levels of MAK-1 and MAK-2 in the non-stressed wild type sample were set as 100% for the quantitative analysis.

We were also interested in assessing the ability of the mutants to activate the MAPK pathways in response to stress-inducing conditions during vegetative growth. Peroxidase treatment has been used as one way to activate MAK-1 and MAK-2 in vegetative cells, and we used peroxidase treatment to evaluate the activation of the MAP kinase pathways in our mutants. Before peroxidase treatment, the MAK-1 and MAK-2 phosphorylation status in mutant vegetative hyphae was similar to the MAK-1 and MAK-2 phosphorylation status in mutant germ tubes/CATs (Figures 8, 9A and 9B) for *Δham-6*, *Δham-7*, *Δamph-1* and *Δwhi-2*. In *Δham-8* and *Δham-9*, the MAK-1 and MAK-2 phosphorylation levels in vegetative hyphae were below the threshold for detection. Stress-induction dramatically increased both MAK-1 and MAK-2 phosphorylation levels in wild type hyphae (Figures 9A and 9B). MAK-1 activation in *Δham-6*, *Δham-7*, *Δham-9*, and *Δwhi-2* was strongly reduced. *Δham-8* showed an intermediate level of MAK-1 activation, while the MAK-1 activation in *Δham-10* and *Δamph-1* was not significantly affected (Figure 9A).

In these experiments with vegetative hyphae, MAK-2 was activated to much lower levels in *Δham-6* and *Δham-9* than in wild type hyphae (Figure 9B). *Δham-8*, *Δham-10*, *Δamph-1* and *Δwhi-2* showed an intermediate level of MAK-2 activation, while MAK-2 activation was normal in *Δham-7* (Figure 9B). The differences in MAK-1 and MAK-2 phosphorylation status between germ tubes/CATs and vegetative hyphae for some of the mutants suggest that there may be differences in how the two MAP kinase pathways are being regulated during the various stages of the *N. crassa* life cycle.

### MAK-1 and MAK-2 nuclear accumulation is normal in mutant germ tubes/CATs

Mutants of the STRIPAK complex have been shown to have a defect in MAK-1 nuclear accumulation, which is regulated by MAK-2 phosphorylation of MOB-3 [34]. MAK-2 nuclear localization is also required during cell fusion [23]. In order to examine if MAK-1 or MAK-2 nuclear accumulation is compromised in the cell fusion mutants, propidium iodide was used to label nuclei in MAK-1-GFP and MAK-2-GFP-expressing mutant strains (Figures S6 and S7). We found that nuclear accumulation of MAK-1 and MAK-2 was normal in all of the cell fusion mutants.

## Discussion

Our screen of approximately 11,000 single gene deletion strains from the first 120 plates of the *N. crassa* single gene deletion library identified 25 genes required for cell-to-cell fusion. This screen identified the MAK-1 and MAK-2 MAPK pathways as well as the STRIPAK complex as three major signaling modules regulating cell fusion [1], [6], [7], [24], [34], [60]. In this report, we focused our research on seven cell fusion genes whose functions were less well-characterized.

HAM-6, HAM-7 and HAM-8 are highly conserved proteins present in all filamentous ascomycetes. The corresponding mutants share the same protoperithecium-deficient, flat conidiation phenotype and are morphologically indistinguishable from each other (Figure 1). They produce abundant macroconidia, but the germinating macroconidia rarely produce CAT-like structures under CAT induction conditions. The *ham-6* gene encodes a 145-amino-acid protein, the *ham-7 gene* encodes a 230-amino-acid protein, and the *ham-8* gene encodes a 597-amino-acid protein. HAM-6 and HAM-8 were predicted to be membrane proteins with 3 and 4 transmembrane domains respectively. HAM-7 is a GPI-anchored cell wall protein, and we have previously shown that it functions as a MAK-1 pathway sensor during hyphal fusion [31]. We found that the three proteins were expressed at much higher levels in germ tubes/CATs than in vegetative hyphae (Figure 3). HAM-7 and HAM-8 were found to be localized in punctate pattern, suggestive of small vesicular or vacuolar structures (Figures 5A and 5B). The HAM-8 containing structures were found to be concentrated near the tip of the germlings and CATs (Figure 5B). Although HAM-7 has been shown to be a GPI-anchored cell wall protein, we did not see immunolocalization of HAM-7 at the plasma membrane/cell wall boundary. We attribute this to the heavily glycosylated status of HAM-7, which could block to interaction between the glycosylated HAM-7 and the antibody used for immunolocalization. The HAM-7 observed in our localization studies (Figure 5A) may well represent newly synthesized HAM-7 in transit through the secretory pathway that hasn’t been fully glycosylated. Tip localization of MAK-2-GFP and SO-GFP was missing in the few CAT-like structures formed by these mutants and the mutants failed to fuse with wild type conidia (Figures 6 and 7). We found that HAM-6, HAM-7 and HAM-8 are required for MAK-1 kinase activation during conidial germination and CAT formation (Figure 8). Despite the lower levels of expression for HAM-6, HAM-7 and HAM-8 in vegetative cells, MAK-1 phosphorylation was dramatically reduced in *Δham-6*, *Δham-7*, and *Δham-8* during vegetative hyphal growth. Leeder et al. [1] determined that the expression of the three genes is co-regulated and controlled by the MAK-2 pathway-dependent transcription factor PP-1. In summary, we propose that the GPI-anchored cell wall HAM-7 and the two transmembrane proteins, HAM-6 and HAM-8, function together to regulate the MAK-1 pathway. Given the cell wall/plasma membrane location for the GPI-anchored protein HAM-7, we suggest that the three proteins might participate in a signaling complex at the cell wall/plasma membrane boundary, but our data would also be consistent with a signaling complex localized to intracellular membranes.

The *ham-9* gene encodes an 869-amino-acid protein containing a SAM domain and two PH domains. The SAM domain has been identified in yeast Ste11p (*S. cerevisae* homolog of *N. crassa* NRC-1) [61], and the PH domains have been suggested to play a role in targeting signal transduction proteins to intracellular membrane in signaling events [62]. The C-terminal GFP-tagged and N-terminal RFP-tagged HAM-9 fusion proteins were not functional, precluding any live-imaging analysis. HA-HAM-9 was expressed in both vegetative hyphae and germ tubes/CATs (Figure 3), but its expression level was too low for immunolocalization. The requirement of HAM-9 for both MAK-1 and MAK-2 activation in vegetative hyphae (Figures 9A and 9B), may suggest that HAM-9 regulates cross-communication of the two MAPK pathways during vegetative growth.

The *amph-1* gene encodes a 262-amino-acid protein containing a bar domain, a domain frequently involved in protein-protein interaction and regulation of membrane curvature [63]. *N. crassa* AMPH-1 is a homolog of the yeast Rvs161p and Rvs167p proteins. Rvs161p and Rvs167p are required for endocytosis and cell fusion during yeast mating [63], [64]. HA-AMPH-1 and RFP-AMPH-1 localized to small vesicles in the germ tubes/CATs, and some of the vesicles appeared to be associated with the plasma membrane (Figures 4 and 5C), suggesting that *N. crassa* AMPH-1 plays a role in vesicular trafficking and endocytosis. MAK-1 and MAK-2 activity in *Δamph-1* germlings were similar to the wild type control, indicating that AMPH-1 does not affect cell fusion by regulating these pathways. This is consistent with our observation that a few *Δamph-1* conidia having wild type morphology were able to participate in cell fusion with wild type conidia (Figure 7). HA-AMPH-1 was expressed in both vegetative hyphae and germlings, suggesting it is a general factor required for all stages of *N. crassa* life cycle. In summary, we suggest that AMPH-1 functions during vesicular trafficking and endocytosis.

The *ham-10* gene encodes a 1,422-amino-acid protein containing a C2 domain near the C terminus. C2 domains function as calcium-dependent lipid-binding domains and are thought to be involved in vesicular trafficking, exocytosis, and signal transduction [65]. HAM-10 tagged with RFP at its N terminus fully rescued *Δham-10*, but HAM-10 tagged with GFP at the C terminus did not (Figures 1 and 2), suggesting that modification near the C terminal C2 domain may affect the function and stability of HAM-10. RFP-HAM-10 localized in the cytosol and in a punctate pattern, suggestive of a vesicular or vacuolar network location (Figure 4). However, our proposed localization of HAM-10 should be considered as a tentative assignment. We did not demonstrate that the RFP-tag remained attached to HAM-10, nor have we carried out extensive co-localization studies with known vesicle and vacuolar marker proteins to definitively demonstrate co-localization of the RFP-HAM-10 with organelle-specific markers. Our results demonstrate that HAM-10 was not enriched at CAT tips during cell fusion (Figures 4 and S3). The requirement of HAM-10 in both MAPK pathways during different development stages suggests HAM-10 could be a general factor in regulating cell growth.

The *whi-2* gene encodes a 298-amino-acid protein with homology to the yeast general stress response protein Whi2p, which has been shown to activate autophagy and mitophagy under nutrient starvation conditions [66], [67]. *N. crassa Δwhi-2* displayed a conidial development defect (Figure S1). HA-WHI-2 was expressed in both vegetative hyphae and germ tubes/CATs and was localized in cytosol and in a punctate pattern suggestive of small vesicles or vacuoles (Figures 3 and 5D). MAK-2/SO signaling was abolished in *Δwhi-2* (Figure 6), but we found that a few mutant macroconidia with wild type morphology were able to participate in cell fusion with wild type conidia (Figure 7). Interestingly, the phosphorylation levels of both MAK-1 and MAK-2 were reduced in germlings and during vegetative hyphal growth (Figures 8 and 9), suggesting WHI-2 functions as a general stress response factor regulating both MAPK pathways.

**Figure 10:**
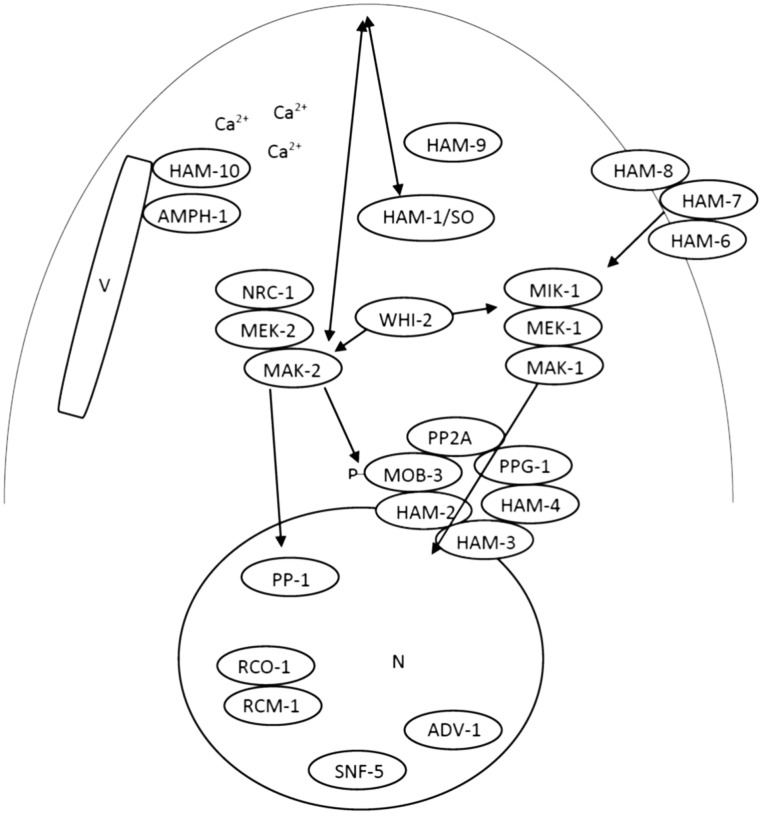
Schematic model for the regulatory network involved in CAT fusion. PP-1, ADV-1, SNF-5, and RCO-1/RCM-1 are transcription factors required for CAT fusion. MIK-1/MEK-1/MAK-1 and NRC-1/MEK-2/MAK-2 are two MAP kinase pathways required for CAT fusion. HAM-2/HAM-3/HAM-4/MOB-3/PP2A/PPG-1 form the STRIPAK complex that regulates MAK-1 nuclear accumulation. HAM-1/SO and MAK-2 engage in Ping-Pong signaling behavior during CAT fusion. HAM-6/HAM-7/HAM-8 are required at the plasma membrane/cell wall for MAK-1 pathway activation. HAM-10 may regulate vesicular trafficking and could potentially respond to calcium signaling during cell fusion. AMPH-1 regulates vesicular trafficking and endocytosis during cell fusion. WHI-2 may regulate the MAP kinase pathways through a general stress response pathway. The role of HAM-9 during CAT fusion remains to be determined.

In summary, our studies on the seven cell fusion genes confirmed that the MAK-1 and the MAK-2 pathways play critical roles during conidia germination and CAT fusion. Figure 10 shows a diagrammatic representation of a CAT tip with many of the proteins we have discussed. The phenotypic characteristics, cell type-specific expression patterns, cellular locations, and MAP kinase activity status of HAM-6, HAM-7 and HAM-8 suggest that the three proteins may form a multimeric sensor complex at the cell wall/plasma membrane or on intracellular vesicles and regulate MAK-1 activation during CAT fusion. Our studies on HAM-9, HAM-10, AMPH-1 and WHI-2 suggest that cell fusion is also affected in mutants lacking proteins with general functions in growth and development. HAM-10, AMPH-1 and WHI-2 clearly play a role in conidial development as well as during CAT fusion. The importance of HAM-9, HAM-10 and WHI-2 for both MAK-1 and MAK-2 signaling may provide opportunities to study cross-talk regulation between MAP kinase pathways and other signaling modules.

## Supporting Information

Figure S1 **CAT fusion in wild type and mutants.** Wild type (WT) and mutant conidia cells were grown under CAT induction conditions for 4 hours. Images for *Δham-6*, *Δham-7*, *Δham-8*, *Δham-9*, *Δham-10*, *Δamph-1*, *Δwhi-2*, and wild type are shown. The images show that conidia from the mutant isolates are unable to generate CATs. The wild type conidia participate in CAT formation and fusion. The arrows in the *Δham-10*, *Δamph-1*, and *Δwhi-2* panels point to chains of abnormal conidia. The arrows in the wild type panel point to a site of CAT fusion.
(TIF)

Figure S2 **Localization of HAM-8-GFP, RFP-HAM-8, and RFP-VAM-3.** Confocal microscopic images were taken of cells expression GFP- and RFP-tagged proteins. A) Images for germ tubes/CATs expressing HAM-8-GFP (top row of panels) and germ tubes/CATs expressing RFP-HAM-8 (bottom row of panels). Fluorescent images (left column), DIC images (middle column), and merged images (right column) are shown. B) Confocal microscopic images were taken for cells expressing both HAM-8-GFP and RFP-VAM-3. GFP fluorescent image (HAM-8-GFP localization in top left panel), DIC image (top right panel), RFP fluorescent image (RFP-VAM-3 localization in bottom left panel), and a merged image (bottom right panel) are shown. Yellow fluorescent signal in the merged image shows co-localization of HAM-8-GFP and RFP-VAM-3.
(TIF)

Figure S3 **Localization of RFP-HAM-10 with SO-GFP.** Heterokaryotic conidia expressing RFP-HAM-10 and SO-GFP were grown under CAT induction conditions for 4 hours. Confocal microscopic images were taken for CATs engaging in cell fusion. GFP fluorescent image (SO-GFP localization in top left panel), DIC image (top right panel), RFP fluorescent image (RFP-HAM-10 localization in bottom left panel), and a merged image (bottom right panel) are shown. The arrows in the fluorescent images point to a site of cell fusion. Note the presence of SO-GFP and the absence of RFP-HAM-10 at the fusion site.
(TIF)

Figure S4 **Localization of RFP-AMPH-1 with MAK-2-GFP.** Heterokaryotic conidia expressing RFP-AMPH-1 and MAK-2-GFP were grown under CAT induction conditions for 4 hours. Confocal microscopic images were taken for CATs engaging in cell fusion. GFP fluorescent image (MAK-2-GFP localization in top left panel), DIC image (top right panel), RFP fluorescent image (RFP-AMPH-1 localization in bottom left panel), and a merged image (bottom right panel) are shown. The arrows in the fluorescent images point to a site where cell fusion will occur. Note the presence of MAK-2-GFP and the absence of RFP-AMPH-1 at the tip of CATs.
(TIF)

Figure S5 **Ponceau stain for MAK-1 and MAK-2 phosphorylation status in mutant germ tubes/CATs.** Ponceau stain image is shown below the Western blot image for the MAK-1 and MAK-2 phosphorylation status in wild type (WT) and mutant germ tubes/CATs. The Western blot image is found as Figure 8 in the manuscript and the Ponceau stain image is given here to demonstrate equal loading of the samples used in the Western blot.
(TIF)

Figure S6 **Nuclear localization of MAK-1-GFP in mutant germ tubes.** Propidium iodide was used to stain nuclei in MAK-1-GFP-expressing wild type (WT) and mutant germ tubes. The figure shows DIC images, GFP fluorescent images, propidium iodide red fluorescent images, and merged images (from left to right respectively). Images are shown for wild type (WT) (row 1), *Δham-6* (row 2), *Δham-7* (row 3), *Δham-8* (row 4), *Δham-9* (row 5), *Δham-10* (row 6), *Δamph-1* (row 7), and *Δwhi-2* (row 8) germ tubes.
(TIF)

Figure S7 **Nuclear localization of MAK-2-GFP in mutant germ tubes.** Propidium iodide was used to stain nuclei in MAK-2-GFP-expressing wild type (WT) and mutant germ tubes. The figure shows DIC images, GFP fluorescent images, propidium iodide red fluorescent images, and merged images (from left to right respectively). Images are shown for wild type (WT) (row 1), *Δham-6* (row 2), *Δham-7* (row 3), *Δham-8* (row 4), *Δham-9* (row 5), *Δham-10* (row 6), *Δamph-1* (row 7), and *Δwhi-2* (row 8) germ tubes.
(TIF)

Table S1 **Plasmids used in this study.**
(DOCX)

Table S2 **Primers used in this study.**
(DOCX)

## References

[1] Leeder AC, Jonkers W, Li J, Glass NL (2013) Germination and Early Colony Establishment in *Neurospora crassa* Requires a MAP Kinase Regulatory Network. Genetics 195: 883–898. 24037267 10.1534/genetics.113.156984 PMC3813871

[2] Roca MG, Read ND, Wheals AE (2005) Conidial anastomosis tubes in filamentous fungi. FEMS Microbiol Lett 249: 191–198. 16040203 10.1016/j.femsle.2005.06.048

[3] Richard F, Glass NL, Pringle A (2012) Cooperation among germinating spores facilitates the growth of the fungus, Neurospora crassa. Biology Letters 8: 419–422. 22258449 10.1098/rsbl.2011.1141 PMC3367758

[4] Simonin A, Palma-Guerrero J, Fricker M, Glass NL (2012) Physiological significance of network organization in fungi. Eukaryot Cell 11: 1345–1352. 22962278 10.1128/EC.00213-12 PMC3486018

[5] Glass NL, Rasmussen C, Roca MG, Read ND (2004) Hyphal homing, fusion and mycelial interconnectedness. Trends in microbiology 12: 135–141. 15001190 10.1016/j.tim.2004.01.007

[6] Fleissner A, Simonin AR, Glass NL (2008) Cell fusion in the filamentous fungus, Neurospora crassa. Methods Mol Biol 475: 21–38. 18979236 10.1007/978-1-59745-250-2_2

[7] Read ND, Fleissner A, Roca MG, Glass NL (2010) Hyphal fusion. In: Borkovich KA, Ebbole DJ, editors. Cellular and molecular biology of filamentous fungi. Washington, DC.: ASM Press. 260–273.

[8] Roca MG, Arlt J, Jeffree CE, Read ND (2005) Cell biology of conidial anastomosis tubes in Neurospora crassa. Eukaryot Cell 4: 911–919. 15879525 10.1128/EC.4.5.911-919.2005 PMC1140100

[9] Fu C, Iyer P, Herkal A, Abdullah J, Stout A, et al (2011) Identification and characterization of genes required for cell-to-cell fusion in Neurospora crassa. Eukaryot Cell 10: 1100–1109. 21666072 10.1128/EC.05003-11 PMC3165452

[10] Aldabbous MS, Roca MG, Stout A, Huang IC, Read ND, et al (2010) The ham-5, rcm-1 and rco-1 genes regulate hyphal fusion in Neurospora crassa. Microbiology 156: 2621–2629. 20522492 10.1099/mic.0.040147-0 PMC3068686

[11] Fleissner A, Sarkar S, Jacobson DJ, Roca MG, Read ND, et al (2005) The so locus is required for vegetative cell fusion and postfertilization events in Neurospora crassa. Eukaryot Cell 4: 920–930. 15879526 10.1128/EC.4.5.920-930.2005 PMC1140088

[12] Cano-Dominguez N, Alvarez-Delfin K, Hansberg W, Aguirre J (2008) NADPH oxidases NOX-1 and NOX-2 require the regulatory subunit NOR-1 to control cell differentiation and growth in Neurospora crassa. Eukaryot Cell 7: 1352–1361. 18567788 10.1128/EC.00137-08 PMC2519770

[13] Maerz S, Dettmann A, Ziv C, Liu Y, Valerius O, et al (2009) Two NDR kinase-MOB complexes function as distinct modules during septum formation and tip extension in Neurospora crassa. Mol Microbiol 74: 707–723. 19788544 10.1111/j.1365-2958.2009.06896.x PMC4617822

[14] Mahs A, Ischebeck T, Heilig Y, Stenzel I, Hempel F, et al (2012) The essential phosphoinositide kinase MSS-4 is required for polar hyphal morphogenesis, localizing to sites of growth and cell fusion in Neurospora crassa. PLoS One 7: e51454. 23272106 10.1371/journal.pone.0051454 PMC3521734

[15] Schurg T, Brandt U, Adis C, Fleissner A (2012) The Saccharomyces cerevisiae BEM1 homologue in Neurospora crassa promotes co-ordinated cell behaviour resulting in cell fusion. Mol Microbiol 86: 349–366. 22906237 10.1111/j.1365-2958.2012.08197.x

[16] Palma-Guerrero J, Glass NL (2013) LFD-1 is a component of the membrane merger machinery during cell-cell fusion in *Neurospora crassa* 27^th^ Fungal Genetics Conference. Asilomar, CA.

[17] Read ND, Goryachev AB, Lichius A (2012) The mechanistic basis of self-fusion between conidial anastomosis tubes during fungal colony initiation. Fungal Biology Reviews 26: 1–11.

[18] Kothe GO, Free SJ (1998) The isolation and characterization of nrc-1 and nrc-2, two genes encoding protein kinases that control growth and development in Neurospora crassa. Genetics 149: 117–130. 9584090 10.1093/genetics/149.1.117 PMC1460147

[19] Pandey A, Roca MG, Read ND, Glass NL (2004) Role of a mitogen-activated protein kinase pathway during conidial germination and hyphal fusion in Neurospora crassa. Eukaryot Cell 3: 348–358. 15075265 10.1128/EC.3.2.348-358.2004 PMC387641

[20] Li D, Bobrowicz P, Wilkinson HH, Ebbole DJ (2005) A mitogen-activated protein kinase pathway essential for mating and contributing to vegetative growth in Neurospora crassa. Genetics 170: 1091–1104. 15802524 10.1534/genetics.104.036772 PMC1451179

[21] Park G, Pan S, Borkovich KA (2008) Mitogen-activated protein kinase cascade required for regulation of development and secondary metabolism in Neurospora crassa. Eukaryot Cell 7: 2113–2122. 18849472 10.1128/EC.00466-07 PMC2593188

[22] Maerz S, Ziv C, Vogt N, Helmstaedt K, Cohen N, et al (2008) The nuclear Dbf2-related kinase COT1 and the mitogen-activated protein kinases MAK1 and MAK2 genetically interact to regulate filamentous growth, hyphal fusion and sexual development in Neurospora crassa. Genetics 179: 1313–1325. 18562669 10.1534/genetics.108.089425 PMC2475735

[23] Fleissner A, Leeder AC, Roca MG, Read ND, Glass NL (2009) Oscillatory recruitment of signaling proteins to cell tips promotes coordinated behavior during cell fusion. Proc Natl Acad Sci U S A 106: 19387–19392. 19884508 10.1073/pnas.0907039106 PMC2780775

[24] Dettmann A, Illgen J, Marz S, Schurg T, Fleissner A, et al (2012) The NDR kinase scaffold HYM1/MO25 is essential for MAK2 map kinase signaling in Neurospora crassa. PLoS Genet 8: e1002950. 23028357 10.1371/journal.pgen.1002950 PMC3447951

[25] Borkovich KA, Alex LA, Yarden O, Freitag M, Turner GE, et al (2004) Lessons from the genome sequence of Neurospora crassa: tracing the path from genomic blueprint to multicellular organism. Microbiol Mol Biol Rev 68: 1–108. 15007097 10.1128/MMBR.68.1.1-108.2004 PMC362109

[26] Rispail N, Soanes DM, Ant C, Czajkowski R, Grunler A, et al (2009) Comparative genomics of MAP kinase and calcium-calcineurin signalling components in plant and human pathogenic fungi. Fungal Genet Biol 46: 287–298. 19570501 10.1016/j.fgb.2009.01.002

[27] Saito H (2010) Regulation of cross-talk in yeast MAPK signaling pathways. Curr Opin Microbiol 13: 677–683. 20880736 10.1016/j.mib.2010.09.001

[28] Goryachev AB, Lichius A, Wright GD, Read ND (2012) Excitable behavior can explain the “ping-pong” mode of communication between cells using the same chemoattractant. Bioessays 34: 259–266. 22271443 10.1002/bies.201100135

[29] Vogt N, Seiler S (2008) The RHO1-specific GTPase-activating protein LRG1 regulates polar tip growth in parallel to Ndr kinase signaling in Neurospora. Mol Biol Cell 19: 4554–4569. 18716060 10.1091/mbc.E07-12-1266 PMC2575149

[30] Khatun R, Lakin-Thomas P (2010) Activation and localization of protein kinase C in *Neurospora crassa* . Fungal Genet Biol 48: 465–473. 21070858 10.1016/j.fgb.2010.11.002

[31] Maddi A, Dettman A, Fu C, Seiler S, Free SJ (2012) WSC-1 and HAM-7 are MAK-1 MAP kinase pathway sensors required for cell wall integrity and hyphal fusion in Neurospora crassa. PLoS One 7: e42374. 22879952 10.1371/journal.pone.0042374 PMC3411791

[32] Richthammer C, Enseleit M, Sanchez-Leon E, Marz S, Heilig Y, et al (2012) RHO1 and RHO2 share partially overlapping functions in the regulation of cell wall integrity and hyphal polarity in Neurospora crassa. Mol Microbiol 85: 716–733. 22703449 10.1111/j.1365-2958.2012.08133.x

[33] Bloemendal S, Bernhards Y, Bartho K, Dettmann A, Voigt O, et al (2012) A homologue of the human STRIPAK complex controls sexual development in fungi. Mol Microbiol 84: 310–323. 22375702 10.1111/j.1365-2958.2012.08024.x

[34] Dettmann A, Heilig Y, Ludwig S, Schmitt K, Illgen J, et al (2013) HAM-2 and HAM-3 are central for the assembly of the Neurospora STRIPAK complex at the nuclear envelope and regulate nuclear accumulation of the MAP kinase MAK-1 in a MAK-2-dependent manner. Mol Microbiol. 90: 796–812. 10.1111/mmi.12399 24028079

[35] Xiang Q, Rasmussen C, Glass NL (2002) The ham-2 locus, encoding a putative transmembrane protein, is required for hyphal fusion in Neurospora crassa. Genetics 160: 169–180. 11805054 10.1093/genetics/160.1.169 PMC1461943

[36] Simonin AR, Rasmussen CG, Yang M, Glass NL (2010) Genes encoding a striatin-like protein (ham-3) and a forkhead associated protein (ham-4) are required for hyphal fusion in Neurospora crassa. Fungal Genet Biol 47: 855–868. 20601042 10.1016/j.fgb.2010.06.010

[37] Freitag M, Hickey PC, Raju NB, Selker EU, Read ND (2004) GFP as a tool to analyze the organization, dynamics and function of nuclei and microtubules in Neurospora crassa. Fungal Genet Biol 41: 897–910. 15341912 10.1016/j.fgb.2004.06.008

[38] Berepiki A, Lichius A, Shoji JY, Tilsner J, Read ND (2010) F-actin dynamics in Neurospora crassa. Eukaryot Cell 9: 547–557. 20139238 10.1128/EC.00253-09 PMC2863416

[39] Lichius A, Lord KM, Jeffree CE, Oborny R, Boonyarungsrit P, et al (2012) Importance of MAP kinases during protoperithecial morphogenesis in Neurospora crassa. PLoS One 7: e42565. 22900028 10.1371/journal.pone.0042565 PMC3416862

[40] Colot HV, Park G, Turner GE, Ringelberg C, Crew CM, et al (2006) A high-throughput gene knockout procedure for Neurospora reveals functions for multiple transcription factors. Proc Natl Acad Sci USA 103: 10352–10357. 16801547 10.1073/pnas.0601456103 PMC1482798

[41] Freitag M, Selker EU (2005) Expression and visualization of red fluorescent protein (RFP) in *Neurospora crassa* . Fungal Genet Newsl 52: 14–17.

[42] McNally MT, Free SJ (1988) Isolation and characterization of a Neurospora glucose-repressible gene. Curr Genet 14: 545–551. 2977301 10.1007/BF00434079

[43] Margolin BS, Freitag M, Selker EU (1997) Improved plasmids for gene targeting at the *his-3* locus of *Neurospora crassa* by electroporation. Fungal Genet Newsl 44: 34–36.

[44] Kawabata T, Inoue H (2007) Detection of physical interactions by immunoprecipitation of FLAG- and HA tagged proteins expressed at the *his-3* locus in *Neurospora crassa* . Fungal Genet Newsl 54: 5–8.

[45] Honda S, Selker EU (2009) Tools for fungal proteomics: multifunctional neurospora vectors for gene replacement, protein expression and protein purification. Genetics 182: 11–23. 19171944 10.1534/genetics.108.098707 PMC2674810

[46] Chenna R, Sugawara H, Koike T, Lopez R, Gibson TJ, et al (2003) Multiple sequence alignment with the Clustal series of programs. Nucleic Acids Res 31: 3497–3500. 12824352 10.1093/nar/gkg500 PMC168907

[47] Linding R, Russell RB, Neduva V, Gibson TJ (2003) GlobPlot: Exploring protein sequences for globularity and disorder. Nucleic Acids Res 31: 3701–3708. 12824398 10.1093/nar/gkg519 PMC169197

[48] Bowman BJ, Draskovic M, Freitag M, Bowman EJ (2009) Structure and distribution of organelles and cellular location of calcium transporters in Neurospora crassa. Eukaryot Cell 8: 1845–1855. 19801418 10.1128/EC.00174-09 PMC2794220

[49] Tinsley JH, Minke PF, Bruno KS, Plamann M (1996) p150Glued, the largest subunit of the dynactin complex, is nonessential in Neurospora but required for nuclear distribution. Mol Biol Cell 7: 731–742. 8744947 10.1091/mbc.7.5.731 PMC275926

[50] Seiler S, Kirchner J, Horn C, Kallipolitou A, Woehlke G, et al (2000) Cargo binding and regulatory sites in the tail of fungal conventional kinesin. Nat Cell Biol 2: 333–338. 10854323 10.1038/35014022

[51] Roca MG, Lichius A, Read ND (2010) How to analyze and quantify conidial anastomosis tube (CAT)-mediated cell fusion. The Neurospora protocol guide.

[52] Lichius A, Roca MG, Read ND (2010) How to distinguish conidial anastomosis tubes (CATs) from germ tubes, and to discriminate between cell fusion mutants blocked in CAT formation and CAT homing. The *Neurospora* protocol guide 1–6.

[53] Roca MG, Kuo HC, Lichius A, Freitag M, Read ND (2010) Nuclear dynamics, mitosis, and the cytoskeleton during the early stages of colony initiation in Neurospora crassa. Eukaryot Cell 9: 1171–1183. 20207852 10.1128/EC.00329-09 PMC2918927

[54] Hickey PC, Jacobson D, Read ND, Glass NL (2002) Live-cell imaging of vegetative hyphal fusion in *Neurospora crassa* . Fungal Genet Biol 37: 109–119. 12223195 10.1016/s1087-1845(02)00035-x

[55] Maniatis T, Fritsch EF, Sambrook J (1982) Molecular cloning: a laboratory manual. Cold Spring Harbor Laboratory, Cold Spring Harbor, NY.

[56] Hickey PC, Swift SR, Roca MG, Read ND (2004) Live-cell Imaging of Filamentous Fungi Using Vital Fluorescent Dyes and Confocal Microscopy. In: Savidge T, Charalabos P, editors. Methods in Microbiology: Academic Press. 63–87.

[57] Radcliffe PA, Binley KM, Trevethick J, Hall M, Sudbery PE (1997) Filamentous growth of the budding yeast *Saccharomyces cerevisiae* induced by overexpression of the WHi2 gene. Microbiology 143 (Pt 6): 1867–1876. 10.1099/00221287-143-6-1867 9202462

[58] Kaida D, Yashiroda H, Toh-e A, Kikuchi Y (2002) Yeast Whi2 and Psr1-phosphatase form a complex and regulate STRE-mediated gene expression. Genes Cells 7: 543–552. 12090248 10.1046/j.1365-2443.2002.00538.x

[59] Greenwald CJ, Kasuga T, Glass NL, Shaw BD, Ebbole DJ, et al (2010) Temporal and spatial regulation of gene expression during asexual development of Neurospora crassa. Genetics 186: 1217–1230. 20876563 10.1534/genetics.110.121780 PMC2998306

[60] Glass NL, Jacobson DJ, Shiu PK (2000) The genetics of hyphal fusion and vegetative incompatibility in filamentous ascomycete fungi. Annu Rev Genet 34: 165–186. 11092825 10.1146/annurev.genet.34.1.165

[61] Grimshaw SJ, Mott HR, Stott KM, Nielsen PR, Evetts KA, et al (2004) Structure of the sterile alpha motif (SAM) domain of the *Saccharomyces cerevisiae* mitogen-activated protein kinase pathway-modulating protein STE50 and analysis of its interaction with the STE11 SAM. J Biol Chem 279: 2192–2201. 14573615 10.1074/jbc.M305605200

[62] Rebecchi MJ, Scarlata S (1998) Pleckstrin homology domains: a common fold with diverse functions. Annu Rev Biophys Biomol Struct 27: 503–528. 9646876 10.1146/annurev.biophys.27.1.503

[63] Ren G, Vajjhala P, Lee JS, Winsor B, Munn AL (2006) The BAR domain proteins: molding membranes in fission, fusion, and phagy. Microbiol Mol Biol Rev 70: 37–120. 16524918 10.1128/MMBR.70.1.37-120.2006 PMC1393252

[64] Youn JY, Friesen H, Kishimoto T, Henne WM, Kurat CF, et al (2010) Dissecting BAR domain function in the yeast Amphiphysins Rvs161 and Rvs167 during endocytosis. Mol Biol Cell 21: 3054–3069. 20610658 10.1091/mbc.E10-03-0181 PMC2929998

[65] Sutton RB, Davletov BA, Berghuis AM, Sudhof TC, Sprang SR (1995) Structure of the first C2 domain of synaptotagmin I: a novel Ca2+/phospholipid-binding fold. Cell 80: 929–938. 7697723 10.1016/0092-8674(95)90296-1

[66] Leadsham JE, Miller K, Ayscough KR, Colombo S, Martegani E, et al (2009) Whi2p links nutritional sensing to actin-dependent Ras-cAMP-PKA regulation and apoptosis in yeast. J Cell Sci 122: 706–715. 19208759 10.1242/jcs.042424 PMC2720921

[67] Mendl N, Occhipinti A, Muller M, Wild P, Dikic I, et al (2011) Mitophagy in yeast is independent of mitochondrial fission and requires the stress response gene WHI2. J Cell Sci 124: 1339–1350. 21429936 10.1242/jcs.076406

