# Supplementary figures and images for: Characterization of the Neurospora crassa Cell Fusion Proteins, HAM-6, HAM-7, HAM-8, HAM-9, HAM-10, AMPH-1 and WHI-2
Source: PLoS One. 2014 Oct 3;9(10):e107773. doi: 10.1371/journal.pone.0107773 (PMC4184795; doi:10.1371/journal.pone.0107773)

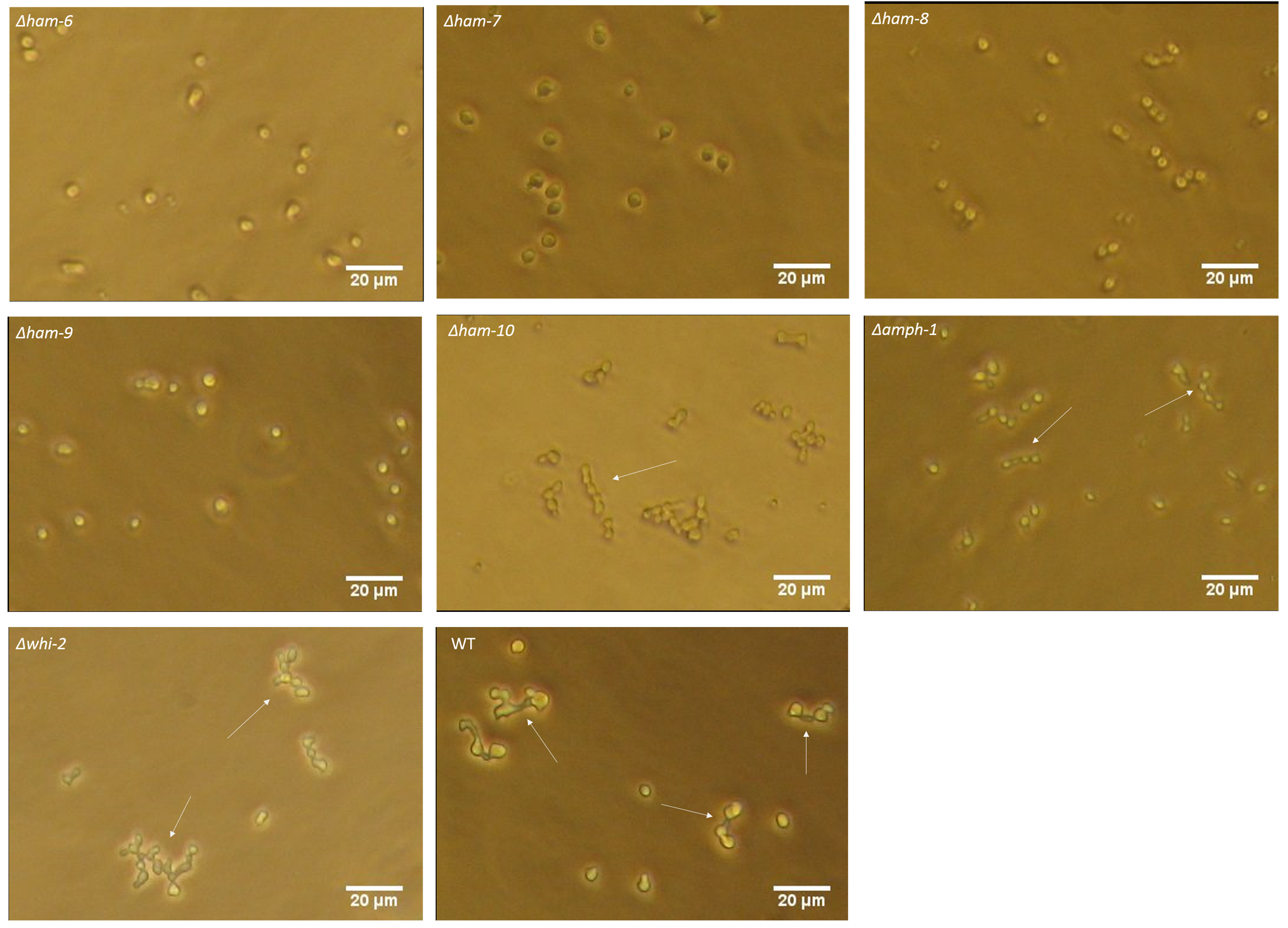

Supplement: Figure S1 — CAT fusion in wild type and mutants. Wild type (WT) and mutant conidia cells were grown under CAT induction conditions for 4 hours. Images for Δham-6, Δham-7, Δham-8, Δham-9, Δham-10, Δamph-1, Δwhi-2, and wild type are shown. The images show that conidia from the mutant isolates are unable to generate CATs. The wild type conidia participate in CAT formation and fusion. The arrows in the Δham-10, Δamph-1, and Δwhi-2 panels point to chains of abnormal conidia. The arrows in the wild type panel point to a site of CAT fusion. (TIF) [file pone.0107773.s001.tif]

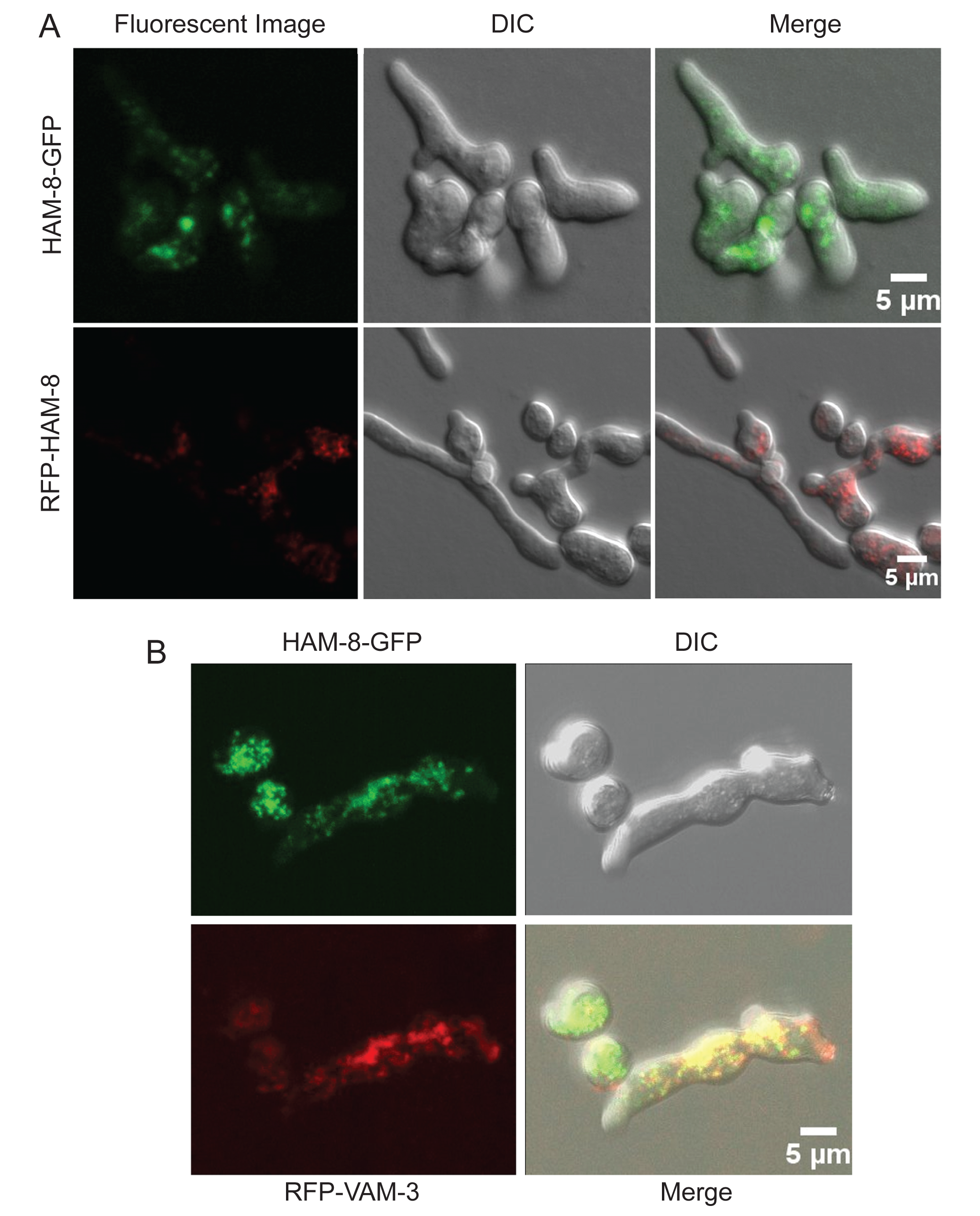

Supplement: Figure S2 — Localization of HAM-8-GFP, RFP-HAM-8, and RFP-VAM-3. Confocal microscopic images were taken of cells expression GFP- and RFP-tagged proteins. A) Images for germ tubes/CATs expressing HAM-8-GFP (top row of panels) and germ tubes/CATs expressing RFP-HAM-8 (bottom row of panels). Fluorescent images (left column), DIC images (middle column), and merged images (right column) are shown. B) Confocal microscopic images were taken for cells expressing both HAM-8-GFP and RFP-VAM-3. GFP fluorescent image (HAM-8-GFP localization in top left panel), DIC image (top right panel), RFP fluorescent image (RFP-VAM-3 localization in bottom left panel), and a merged image (bottom right panel) are shown. Yellow fluorescent signal in the merged image shows co-localization of HAM-8-GFP and RFP-VAM-3. (TIF) [file pone.0107773.s002.tif]

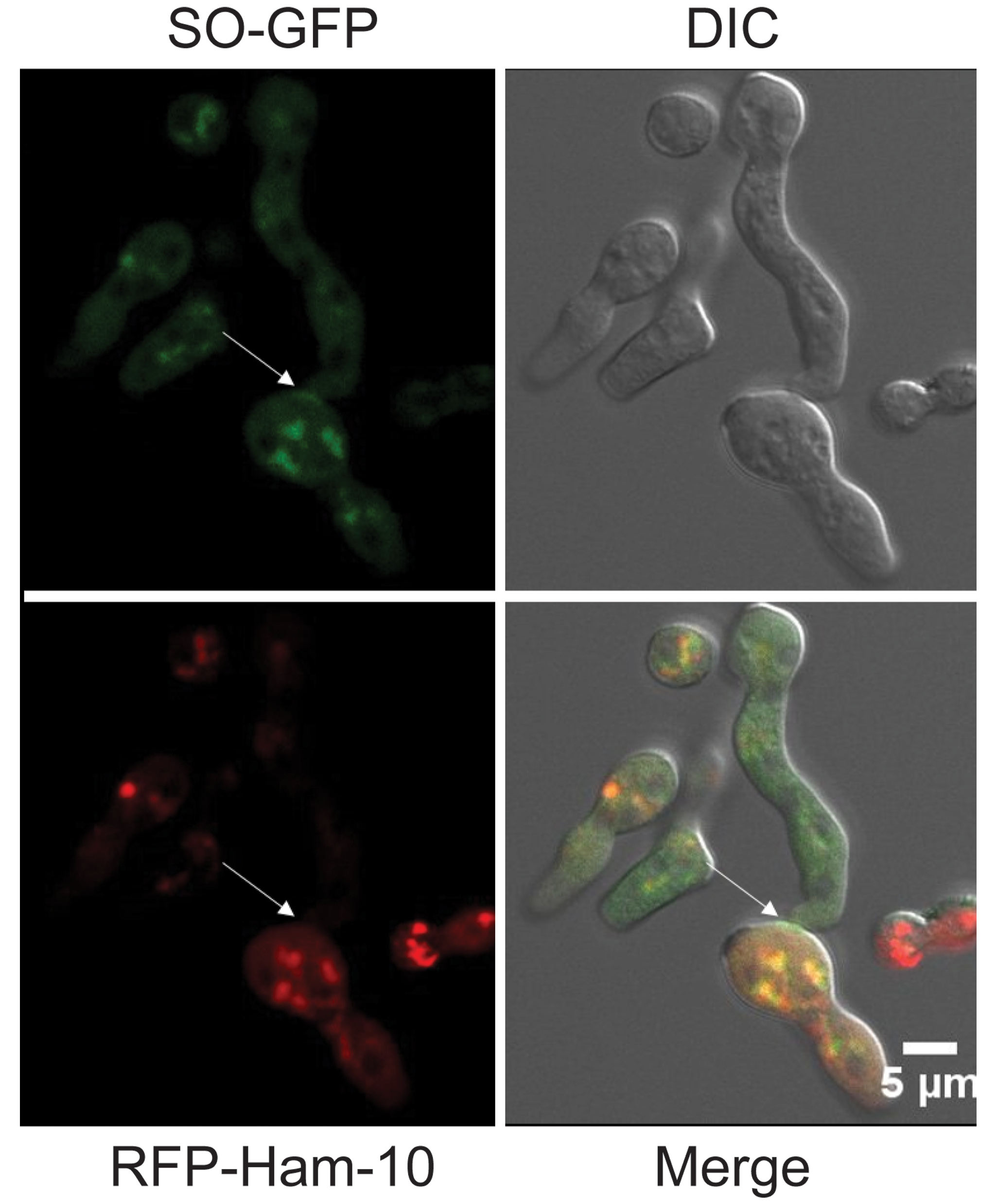

Supplement: Figure S3 — Localization of RFP-HAM-10 with SO-GFP. Heterokaryotic conidia expressing RFP-HAM-10 and SO-GFP were grown under CAT induction conditions for 4 hours. Confocal microscopic images were taken for CATs engaging in cell fusion. GFP fluorescent image (SO-GFP localization in top left panel), DIC image (top right panel), RFP fluorescent image (RFP-HAM-10 localization in bottom left panel), and a merged image (bottom right panel) are shown. The arrows in the fluorescent images point to a site of cell fusion. Note the presence of SO-GFP and the absence of RFP-HAM-10 at the fusion site. (TIF) [file pone.0107773.s003.tif]

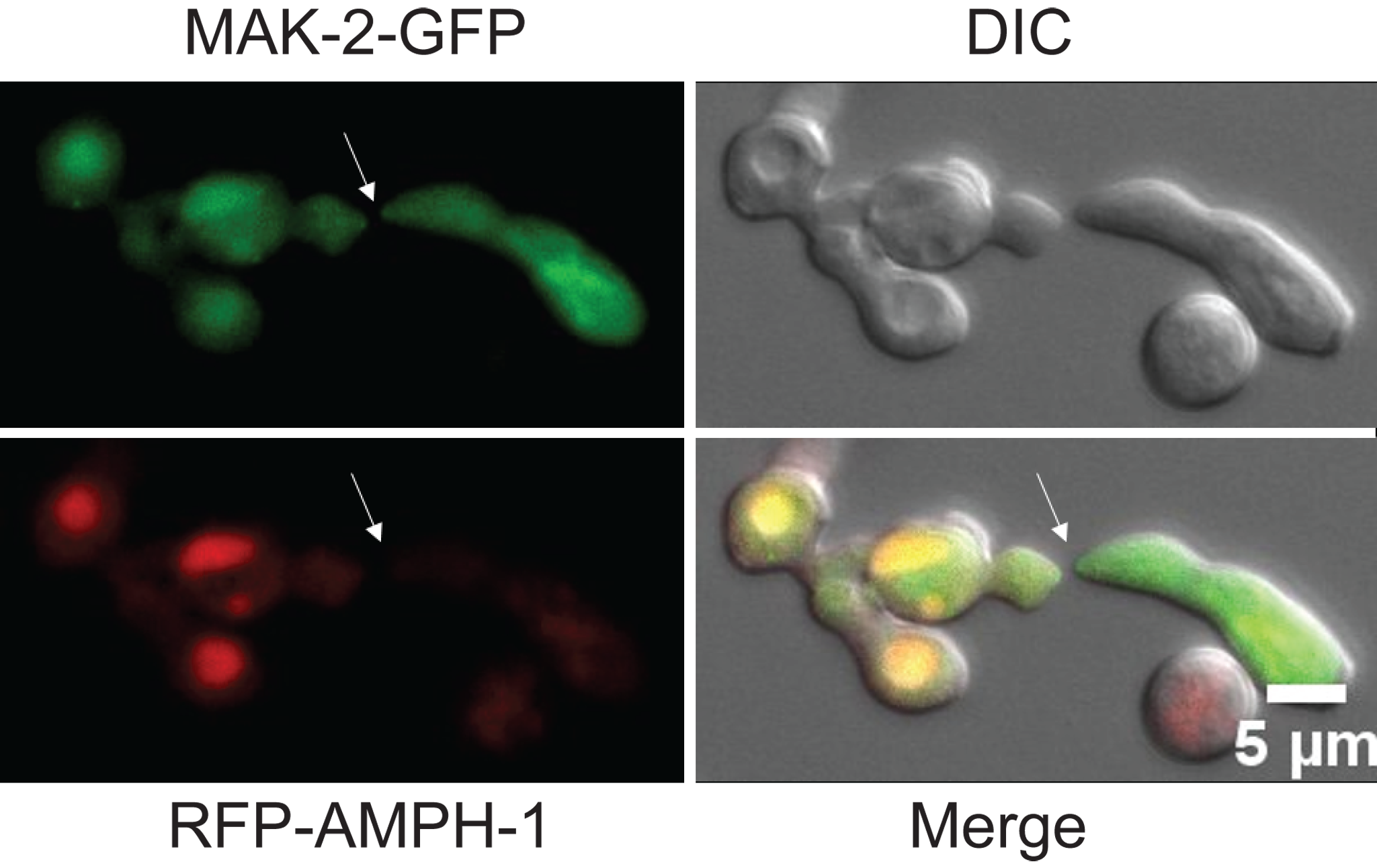

Supplement: Figure S4 — Localization of RFP-AMPH-1 with MAK-2-GFP. Heterokaryotic conidia expressing RFP-AMPH-1 and MAK-2-GFP were grown under CAT induction conditions for 4 hours. Confocal microscopic images were taken for CATs engaging in cell fusion. GFP fluorescent image (MAK-2-GFP localization in top left panel), DIC image (top right panel), RFP fluorescent image (RFP-AMPH-1 localization in bottom left panel), and a merged image (bottom right panel) are shown. The arrows in the fluorescent images point to a site where cell fusion will occur. Note the presence of MAK-2-GFP and the absence of RFP-AMPH-1 at the tip of CATs. (TIF) [file pone.0107773.s004.tif]

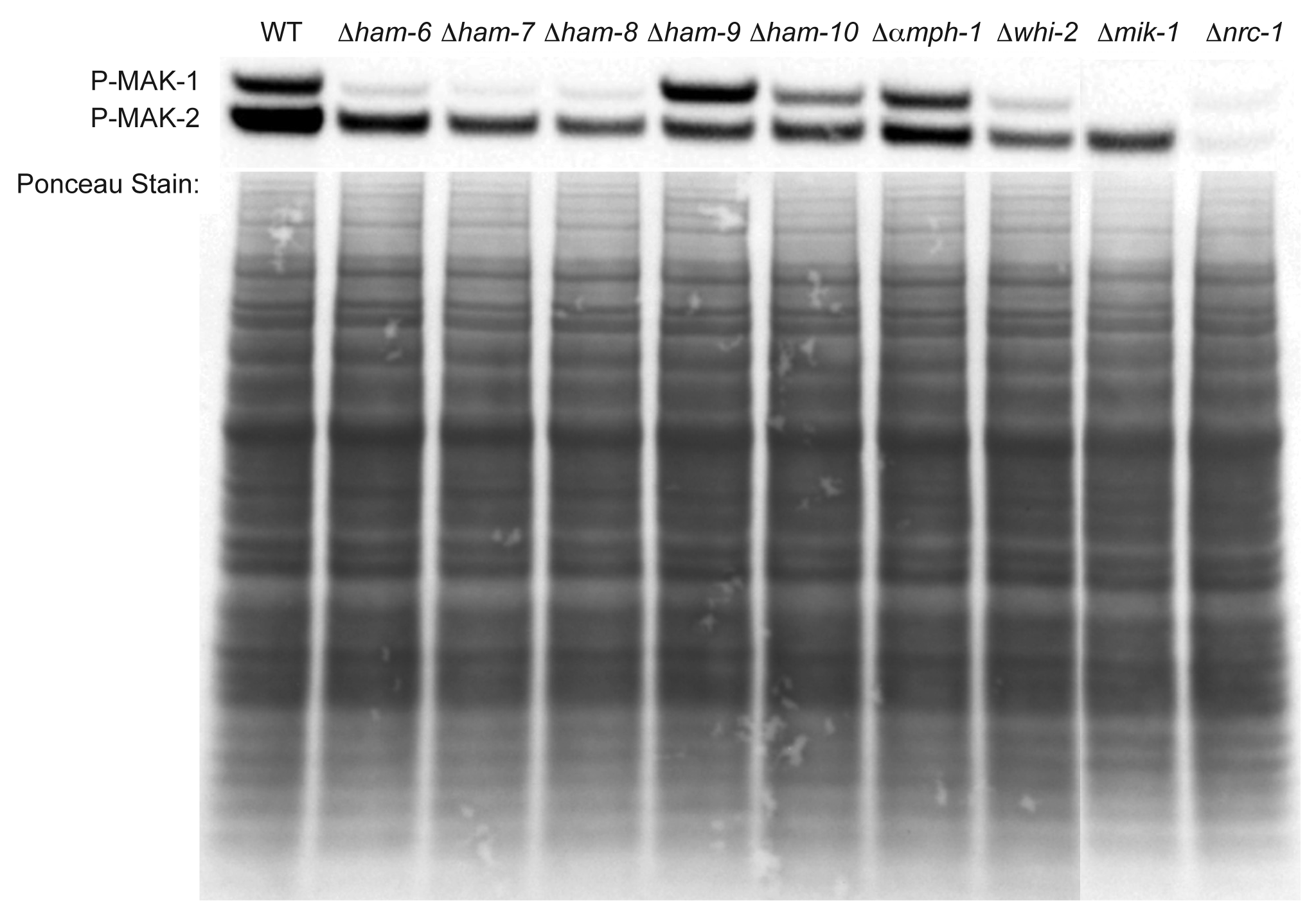

Supplement: Figure S5 — Ponceau stain for MAK-1 and MAK-2 phosphorylation status in mutant germ tubes/CATs. Ponceau stain image is shown below the Western blot image for the MAK-1 and MAK-2 phosphorylation status in wild type (WT) and mutant germ tubes/CATs. The Western blot image is found as Figure 8 in the manuscript and the Ponceau stain image is given here to demonstrate equal loading of the samples used in the Western blot. (TIF) [file pone.0107773.s005.tif]

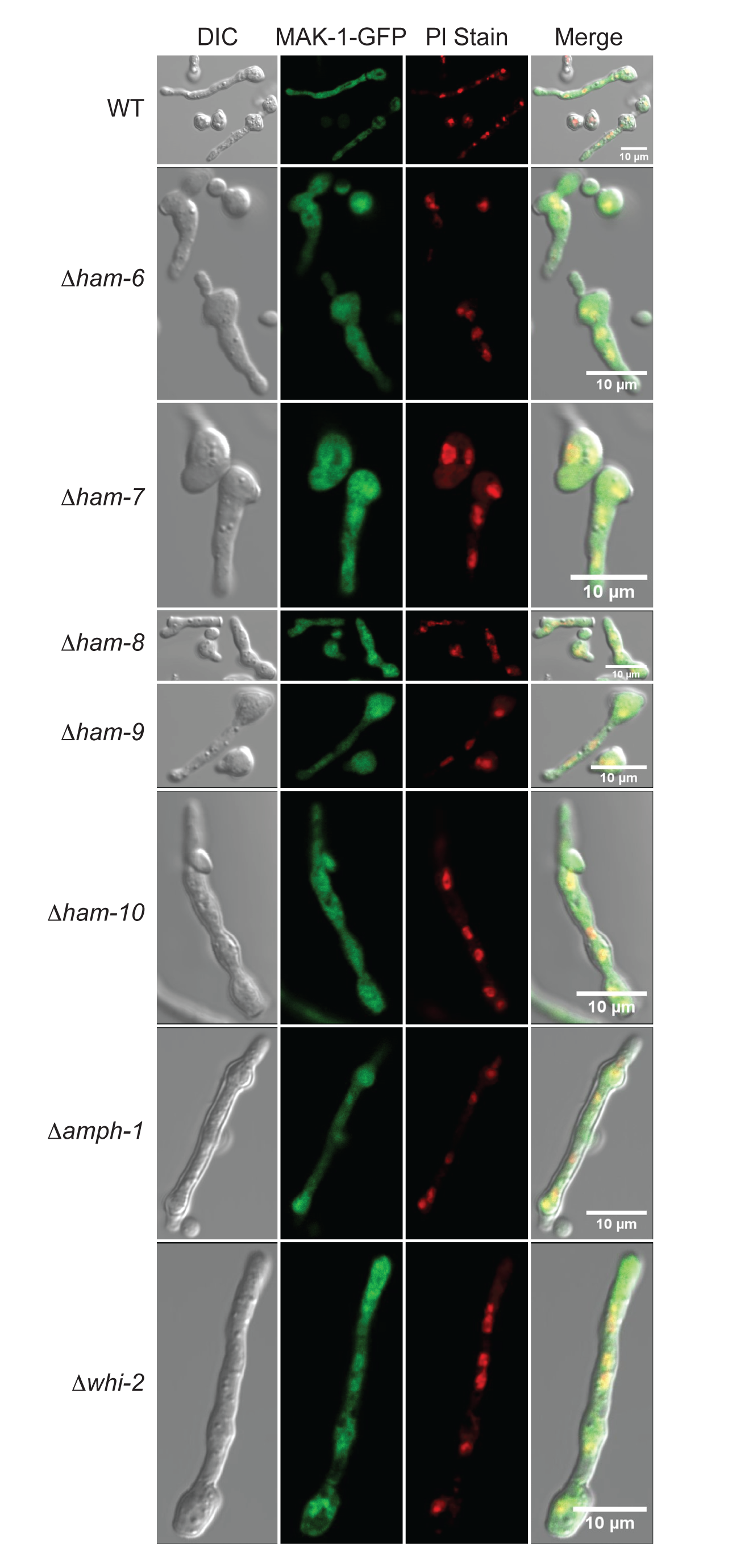

Supplement: Figure S6 — Nuclear localization of MAK-1-GFP in mutant germ tubes. Propidium iodide was used to stain nuclei in MAK-1-GFP-expressing wild type (WT) and mutant germ tubes. The figure shows DIC images, GFP fluorescent images, propidium iodide red fluorescent images, and merged images (from left to right respectively). Images are shown for wild type (WT) (row 1), Δham-6 (row 2), Δham-7 (row 3), Δham-8 (row 4), Δham-9 (row 5), Δham-10 (row 6), Δamph-1 (row 7), and Δwhi-2 (row 8) germ tubes. (TIF) [file pone.0107773.s006.tif]

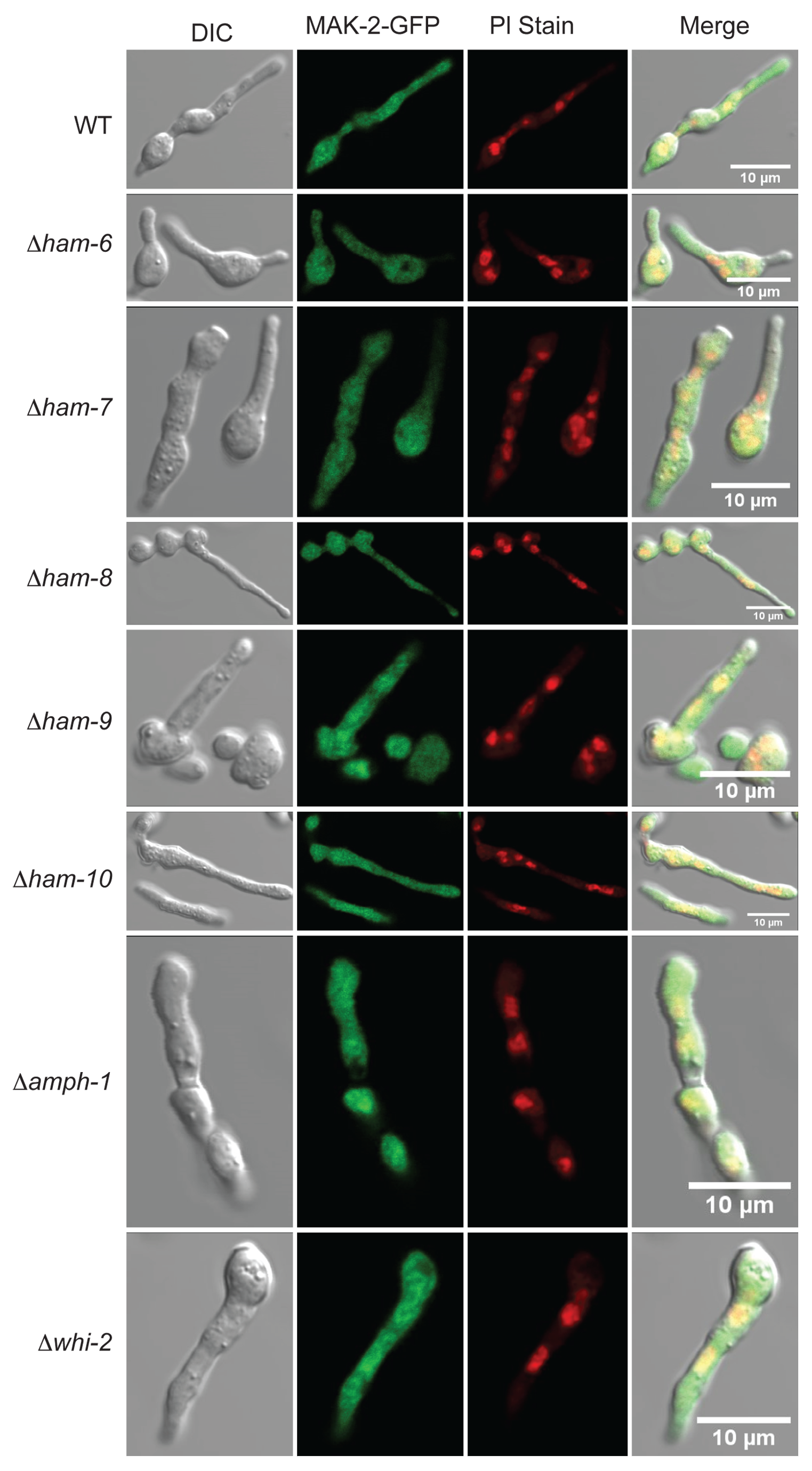

Supplement: Figure S7 — Nuclear localization of MAK-2-GFP in mutant germ tubes. Propidium iodide was used to stain nuclei in MAK-2-GFP-expressing wild type (WT) and mutant germ tubes. The figure shows DIC images, GFP fluorescent images, propidium iodide red fluorescent images, and merged images (from left to right respectively). Images are shown for wild type (WT) (row 1), Δham-6 (row 2), Δham-7 (row 3), Δham-8 (row 4), Δham-9 (row 5), Δham-10 (row 6), Δamph-1 (row 7), and Δwhi-2 (row 8) germ tubes. (TIF) [file pone.0107773.s007.tif]
