# Supplementary material for: Characterization of the Neurospora crassa Cell Fusion Proteins, HAM-6, HAM-7, HAM-8, HAM-9, HAM-10, AMPH-1 and WHI-2
Source: PLoS One. 2014 Oct 3;9(10):e107773. doi: 10.1371/journal.pone.0107773 (PMC4184795; doi:10.1371/journal.pone.0107773)
Supplement: Table S1 — Plasmids used in this study. (DOCX) [file pone.0107773.s008.docx]

**Table S1. Plasmids used in this study.**

| Plasmid | *N. crassa* Gene | Promoter | Host vector | Plasmid source |
| --- | --- | --- | --- | --- |
| pgrp-GFP | NCU03982 | ccg-1 | pMF272 | [47] |
| pRFP-vps-52 | NCU05273 | ccg-1 | pMF334 | [47] |
| parg-4-GFP | NCU10468 | ccg-1 | pMF272 | [47] |
| pRFP-vam-3 | NCU06777 | ccg-1 | pMF334 | [47] |
| pso-GFP | NCU02794 | ccg-1 | pMF272 | [22] |
| pmak-2-GFP | NCU02393 | ccg-1 | pMF272 | [22] |
| pham-8-GFP | NCU02811 | ccg-1 | pMF272 | This study |
| pRFP-ham-8 | NCU02811 | ccg-1 | pMF334 | This study |
| pRFP-ham-10 | NCU02833 | ccg-1 | pMF334 | This study |
| pRFP-amph-1 | NCU01069 | ccg-1 | pMF334 | This study |
| pHA-ham-6 | NCU02767 | ham-6 | pBM60 | This study |
| pHA-ham-7 | NCU00881 | ham-7 | pBM60 | This study |
| pHA-ham-8 | NCU02811 | ham-8 | pBM60 | This study |
| pHA-ham-9 | NCU07389 | ham-9 | pBM60 | This study |
| pHA-amph-1 | NCU01069 | amph-1 | pBM60 | This study |
| pHA-whi-2 | NCU10518 | whi-2 | pBM61 | This study |
