# Supplementary material for: Characterization of the Neurospora crassa Cell Fusion Proteins, HAM-6, HAM-7, HAM-8, HAM-9, HAM-10, AMPH-1 and WHI-2
Source: PLoS One. 2014 Oct 3;9(10):e107773. doi: 10.1371/journal.pone.0107773 (PMC4184795; doi:10.1371/journal.pone.0107773)
Supplement: Table S2 — Primers used in this study. (DOCX) [file pone.0107773.s009.docx]

**Table S2. Primers used in this study.**

| Construct | Primer sequences |
| --- | --- |
| HA-HAM-6 EcoRI-F | ATATGAATTCGGAAGCACGATGCGTCAACC |
| HA-HAM-6 HA-R | GGCGTAGTCGGGGACGTCGTAGGGGTATACACGTCCAGTCTTGCCACC |
| HA-HAM-6 HA-F | TACCCCTACGACGTCCCCGACTACGCCTGATATCCATGATGCTACATATC |
| HA-HAM-6 NotI-R | AATTGCGGCCGCCCACATGCAAGTCTGACAT |
| HA-HAM-8 SpeI-F | ATTAACTAGTCGCGGAGAAGAGAGC |
| HA-HAM-8 HA-R | GGCGTAGTCGGGGACGTCGTAGGGGTAAGCAAACCTCGTCTGCATAGG |
| HA-HAM-8 HA-F | TACCCCTACGACGTCCCCGACTACGCCTAGGTGGTGGTCACCGGGTTC |
| HA-HAM-8 NotI-R | TTTAGCGGCCGCGTGTTCCCGCTTGGTGATTG |
| HA-HAM-9 EcoRI-F | GACGGAACAGAATGGCAGTTG |
| HA-HAM-9 HA-R | GGCGTAGTCGGGGACGTCGTAGGGGTAAATCATGCTGCCATTGACAAC |
| HA-HAM-9 HA-F | TACCCCTACGACGTCCCCGACTACGCCTGAATGCTTTCTTTCTTTATT |
| HA-HAM-9 XbaI-R | ATTATCTAGACCAGTCTCTGCCTGATTCCC |
| HA-AMPH-1 EcoRI-F | ATTAGAATTCGAGGACAAAGTCGGACACAGTG |
| HA-AMPH-1 HA-R | GGCGTAGTCGGGGACGTCGTAGGGGTACCACGACATGATGGCTGAGTTATTCTC |
| HA-AMPH-1 HA-F | TACCCCTACGACGTCCCCGACTACGCCGGTACGCACAAATCCCAC |
| HA-AMPH-1 XbaI-R | ATTATCTAGACCGACAGGACCAACGAATGAGAC |
| HA-WHI-2 NotI-F | TTATGCGGCCGCGATTCTGGACCGTTCACTTCC |
| HA-WHI-2 HA-R | TGCGTAGTCGGGGACGTCGTAGGGGTATATGCCGTCACTGCCATCTTG |
| HA-WHI-2 HA-F | TACCCCTACGACGTCCCCGACTACGCAAGGGGCGATGACGGTTCTAAG |
| HA-WHI-2 SpeI-R | TTTAACTAGTGCTGCGAGTTCTCTGTCGAG |
| HAM-8-GFP F | GGTTTCTAGACAACATGGCCTTTAACGACGAG |
| HAM-8-GFP R | GACGTTAATTAACCTCGTCTGCATAGGCGG |
| RFP-HAM-8 -F | TCCTAGATCTATGGCCTTTAACGACGAGAAG |
| RFP-HAM-8-R | GAGTTCTAGACTAAGCAAACCTCGTCTGCATAGG |
| RFP-HAM-10-F | TGCTGGCCGGCCAATGTCGGGGTCTACGCGACAC |
| RFP-HAM-10-R | TGAATCTAGATCACCTCCCGCCCCTCCCAAAACTG |
| RFP-AMPH-1-F | TGCTAGATCTATGTCGTGGGCCGGTACGCAC |
| RFP-AMPH-1-R | GAGATCTAGATTAAACAGTTCCGCTGATACTCAG |
